# MAF-SleepNet: A Multimodal Attention-Enhanced Fusion Network for Automatic Multi-Class Sleep Disorder Classification from Polysomnography

**DOI:** 10.3390/diagnostics16152317

**Published:** 2026-07-23

**Authors:** Suleyman Yaman, Hasan Guler, Abdul Hafeez-Baig

**Affiliations:** 1 Department of Biomedical, Vocational School of Technical Sciences, Firat University, 23119 Elazig, Türkiye; 2 School of Mathematics, Physics, and Computing, University of Southern Queensland, Springfield, QLD 4300, Australia; 3 Department of Electrical-Electronics Engineering, Faculty of Engineering, Firat University, 23119 Elazig, Türkiye; hasanguler@firat.edu.tr; 4 School of Business, University of Southern Queensland, Toowoomba, QLD 4350, Australia; abdul.hafeez-baig@unisq.edu.au

**Keywords:** attention mechanism, deep learning, information fusion, multimodal learning, polysomnography, sleep disorder classification

## Abstract

**Background/Objectives:** Sleep disorders are heterogeneous conditions with diverse neural, muscular, and ocular manifestations, making polysomnography (PSG) the gold standard for accurate diagnosis. Artificial intelligence-based approaches, particularly deep learning (DL) models capable of integrating heterogeneous information, offer a promising solution for reliable decision-making in such clinical scenarios. However, most existing DL studies have focused on a single disorder, relied on limited datasets, or employed epoch-level labeling strategies that overlook the episodic nature of sleep pathophysiology, thereby limiting clinical applicability. To address these gaps, we propose a novel multimodal attention-enhanced fusion network (MAF-SleepNet) for automatic multi-class sleep disorder classification based on the International Classification of Sleep Disorders. **Methods:** MAF-SleepNet jointly processes electroencephalography (EEG), electrooculography (EOG), and leg electromyography (EMG) signals through modality-specific feature extraction and adaptive attention mechanisms, capturing both intra- and inter-modality dependencies. The model was evaluated on a combined dataset of 141 recordings from three public databases, including five PSG-requiring disorders and a healthy class. **Results:** Experimental results demonstrated that MAF-SleepNet achieved 86.07 ± 3.66% accuracy and 82.67 ± 4.46% macro-F1 under a strict subject-independent cross-validation, and 98.96 ± 0.66% accuracy and 98.93 ± 0.60% macro-F1 under subject-dependent cross-validation. **Conclusions:** These results demonstrate that the proposed approach provides a more reliable and clinically meaningful assessment compared to many existing studies that rely on subject-dependent evaluation or epoch-level labeling. The findings highlight the effectiveness of adaptive multimodal fusion for robust and clinically relevant sleep disorder classification. Future work should investigate the integration of respiratory and autonomic modalities and validation on larger multi-center cohorts.

## 1. Introduction

Sleep is a fundamental biological process that occupies approximately one-third of the human lifespan and plays a critical role in maintaining cognitive, emotional, and physiological functions [1,2,3,4]. Inadequate or poor-quality sleep impairs attention, memory, emotional regulation, and decision-making while increasing the risk of cardiovascular, metabolic, and mental health problems [2,5,6]. Although major organizations recommend at least 7 h of sleep per night for adults [5,7], epidemiological evidence indicates that approximately one-third of adults fail to meet these recommendations, often due to lifestyle factors and, importantly, underlying sleep disorders [3,4,8,9]. These consequences highlight the clinical and public health importance of accurate sleep disorder diagnosis and effective management.

**Figure 1:**
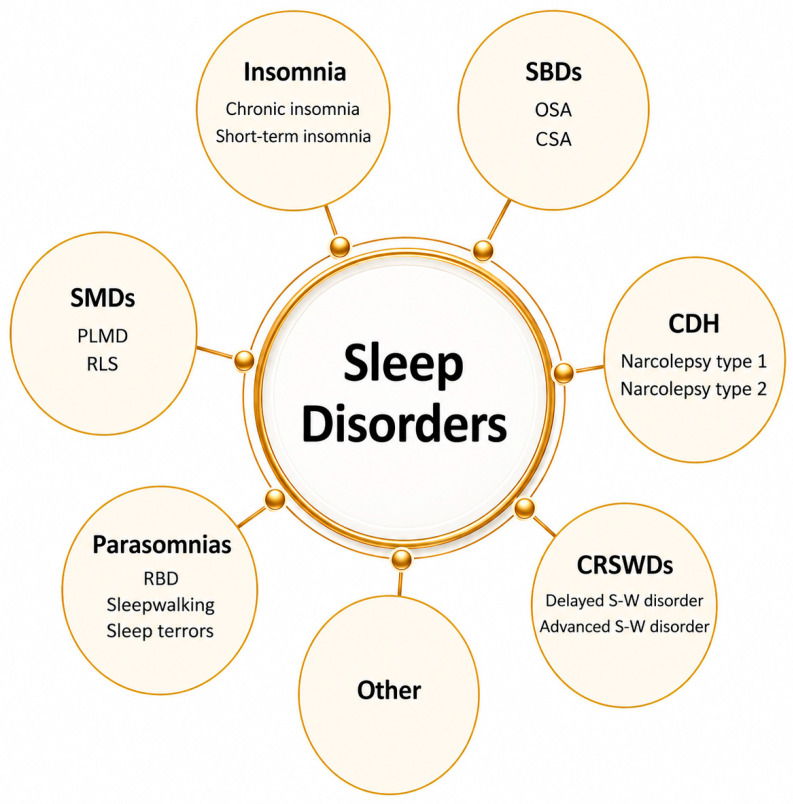
ICSD-3 [10] classification of sleep disorders with representative examples for each category. CDH: central disorders of hypersomnolence; CRSWDs: circadian rhythm sleep–wake disorders; CSA: central sleep apnea; OSA: obstructive sleep apnea; PLMD: periodic limb movement disorder; RBD: REM sleep behavior disorder; RLS: restless legs syndrome; SBDs: sleep-related breathing disorders; SMDs: sleep-related movement disorders; S-W: sleep–wake.

Sleep disorders are a heterogeneous group of conditions that disrupt the quantity, quality, or timing of sleep, leading to impaired daytime functioning and adverse health consequences. These disorders may arise from various physiological, behavioral, and environmental factors [10,11]. The most widely adopted classification framework, the International Classification of Sleep Disorders, Third Edition (ICSD-3) [10], categorizes sleep disorders into seven major categories: insomnia, sleep-related breathing disorders (SBDs), central disorders of hypersomnolence (CDH), circadian rhythm sleep–wake disorders (CRSWDs), parasomnias, sleep-related movement disorders (SMDs), and other sleep disorders, as illustrated in Figure 1. This diversity increases the complexity of accurate diagnosis and highlights the need for reliable and scalable diagnostic approaches.

Polysomnography (PSG) is considered the gold-standard diagnostic tool for the detection of sleep disorders, as it provides a comprehensive assessment of physiological activity during sleep. It involves the simultaneous recording of multiple physiological signals (e.g., electroencephalography (EEG), electrooculography (EOG), chin and leg electromyography (EMG)) [12,13,14,15,16]. However, the clinical interpretation of PSG recordings relies on manual scoring procedures performed by trained experts in accordance with standardized guidelines, such as those of the AASM [17,18]. This process typically requires detailed inspection of long-duration overnight recordings across multiple signal channels to identify sleep stages and transient events such as arousals, limb movements, and respiratory disturbances, making the process time-consuming and labor-intensive [6,19,20,21]. Furthermore, despite standardized criteria, inter-scorer variability remains a challenge, as differences in expert interpretation can affect diagnostic consistency [22,23]. These limitations contribute to diagnostic delays, increased costs, and restricted accessibility in clinical practice. Therefore, developing automated approaches for reliable sleep disorder classification from PSG data is essential to improve efficiency, scalability, and clinical applicability [6,21].

In recent years, artificial intelligence (AI), particularly deep learning (DL), has achieved remarkable success in various domains such as biomedical data processing [24,25,26,27], natural language processing [28,29], and geoscience [30], with a growing number of applications in sleep medicine, including sleep staging [31,32,33], Cyclic Alternating Pattern (CAP) scoring [34,35,36], and diagnosis of sleep disorders [6,21,37]. Despite these advances, several important limitations remain in existing automatic sleep disorder classification studies. Most prior works have focused on single-disorder classification, particularly obstructive sleep apnea (OSA), which limits their ability to address the broader spectrum of clinically relevant sleep disorders defined in the ICSD-3 [6,21].

More recent studies have extended this task to multi-class classification, enabling the differentiation of multiple disorder categories. In these multi-class approaches, a wide range of methods have been explored, including traditional machine learning (ML) techniques based on hand-crafted features [20,38,39,40,41] and DL architectures capable of learning task-specific representations directly from raw or minimally processed PSG signals [19,42,43]. Despite these advances, important limitations still hinder clinical applicability. One major challenge is the inherently episodic nature of sleep disorders, which manifest only during specific sleep stages or time intervals rather than continuously throughout the night [10,44,45,46,47]. However, nearly all existing multi-class classification studies [19,20,38,39,40,42,43]—except for one [41]—rely on epoch-level labeling, where each segment is assigned the subject-level diagnosis regardless of whether pathological events are actually present. Consequently, models may learn subject-specific patterns instead of true pathological characteristics, resulting in inflated performance estimates and limited clinical validity. In addition, several studies [19,20,38,39,42,43] employ evaluation strategies that are not subject-independent, allowing data from the same subject to appear in both training and testing sets. This results in data leakage and overly optimistic performance estimates. Furthermore, traditional machine learning approaches [20,38,39,40,41] based on hand-crafted features require complex, expertise-dependent preprocessing and may fail to capture the complex, non-linear relationships present in high-dimensional PSG data [6,21]. Another important limitation is the reliance on a single dataset [19,20,38,39,40,42,43], most commonly the CAP Sleep Database (CAPSD) [48,49], which limits generalizability across populations, recording conditions, and devices. These challenges highlight the need for robust, generalizable, and clinically reliable approaches for automatic sleep disorder classification.

To address the key gaps identified in the literature, we propose a novel feature-driven hybrid DL-based model, Multi-Modal Attention-Enhanced Fusion Network for Sleep Disorder Classification (MAF-SleepNet). The proposed network integrates handcrafted feature extraction with DL-based representation learning to effectively exploit complementary information from multiple PSG modalities within a unified architecture, enabling more comprehensive modeling of physiological sleep patterns. In addition, it incorporates a modality-specific attention mechanism to enhance feature representation and a feature fusion strategy to integrate heterogeneous information across different physiological domains. The resulting multi-modal representation is utilized for the classification of multiple sleep disorder categories defined in the ICSD-3, along with a normal/healthy class. Furthermore, to overcome limitations in prior studies related to single-dataset usage and subject-dependent evaluation, MAF-SleepNet is assessed under a strictly subject-independent setting using data pooled from three PSG datasets, ensuring a more reliable and generalizable performance evaluation. The model demonstrates strong performance under these conditions, highlighting its robustness and generalization capability.

The key novelties and contributions of this study are outlined below:

- To the best of our knowledge, MAF-SleepNet is the first feature-driven hybrid DL architecture for multi-class sleep disorder classification that enables unified modeling of multiple PSG modalities within a single framework.
- A whole-night learning framework is proposed to address the limitations of epoch-level labeling in previous studies, reducing label noise by avoiding the mislabeling of normal epochs and enabling the model to learn from the complete temporal context of PSG recordings.
- This is the first study to incorporate modality-specific attention mechanisms for the multi-class sleep disorder classification task, enabling adaptive focus on the most informative pathological patterns within each modality.
- A systematic analysis of individual PSG modalities, including EEG, EOG, ECG, chin EMG, and leg EMG, and their combinations is performed to identify the most informative modality configurations for multi-class sleep disorder classification.

The remainder of this paper is organized as follows: Section 2 reviews related work on multi-class sleep disorder classification approaches. Section 3 presents the datasets, the preprocessing steps, and the proposed MAF-SleepNet architecture. Section 4 describes the experimental setup and reports the results, including ablation studies. Section 5 provides an in-depth discussion of the results, and Section 6 concludes the paper with future directions.

## 2. Related Studies

Automatic detection of sleep disorders from PSG has been extensively studied using both ML and DL techniques, aiming to reduce expert workload, minimize inter- and intra-observer variability, and improve diagnostic efficiency. However, the vast majority of existing studies have focused on single-disorder classification, particularly obstructive sleep apnea (OSA), due to its high prevalence and well-documented clinical impact [6,21,50]. Recent review studies [6,21] have consistently highlighted this imbalance, showing that other clinically important sleep disorders have received comparatively limited attention. Although these single-disorder approaches have achieved promising performance, their clinical applicability remains limited, as real-world scenarios require distinguishing among multiple sleep disorders. Therefore, developing multi-class classification frameworks that can capture the diversity of disorders defined in the ICSD-3 [10] is essential for clinically viable automated diagnosis systems.

In recent years, a limited but growing body of research has begun to explore multi-class sleep disorder classification to better reflect real-world clinical scenarios. Table 1 summarizes these studies, detailing the methods employed, datasets used, number of subjects, performance metrics achieved, and the limitations. Except for one study [41], the remaining seven existing studies [19,20,38,39,40,42,43] relied on the CAPSD [48,49], which raises concerns regarding generalizability.

These multi-class studies can be broadly categorized into two groups based on the employed methodologies: conventional ML approaches relying on hand-crafted features and DL architectures capable of learning task-specific representations directly from raw or minimally processed PSG signals. Classical ML techniques such as support vector machines (SVMs) [41], random forests (RFs) [40], and decision tree ensembles [20,38,39] have been applied; however, they typically require labor-intensive, expert-driven feature extraction and selection processes. Moreover, such handcrafted features often fail to capture the complex nonlinear relationships and temporal dependencies inherent in PSG data, and the selection of optimal classifiers is frequently based on trial-and-error procedures [6,21].

These limitations have motivated the transition toward DL-based approaches, including convolutional neural networks (CNNs) [19,42], and hybrid architectures with recurrent neural networks (RNNs) [43], which enable automatic and hierarchical feature learning. In particular, the reliance on limited and often homogeneous datasets restricts the generalizability of existing models. In addition, many approaches [19,20,38,39,40,42,43] adopt epoch-level labeling strategies, assigning the subject-level diagnosis to all segments, thereby introducing substantial label noise due to the episodic nature of sleep disorders. Furthermore, the exploration of modality combinations remains limited, and attention mechanisms have not been sufficiently explored within multi-class sleep disorder classification frameworks, restricting the ability to effectively model complex multi-modal physiological interactions. These limitations highlight the need for more robust and generalizable approaches that can integrate diverse PSG modalities and better capture both temporal and cross-modal dependencies.

## 3. Methodology

**Figure 2:**
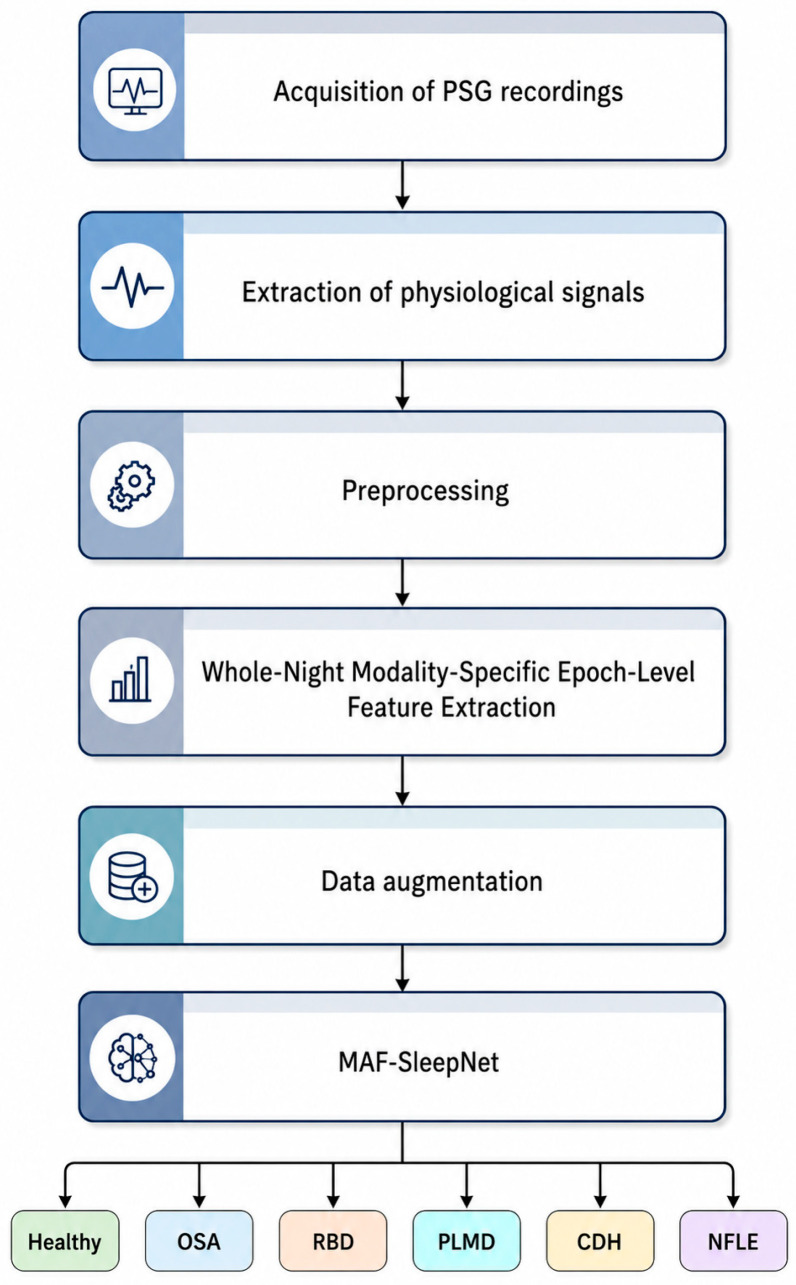
Overall workflow of the proposed methodology for automatic sleep disorder classification.

The overall workflow of the proposed methodology for automatic sleep disorder classification is illustrated in Figure 2. The framework begins with the acquisition of overnight PSG recordings, which are first subjected to standardized preprocessing procedures to ensure signal quality and cross-dataset compatibility. Subsequently, for each physiological modality, a set of domain-specific handcrafted features is extracted from consecutive 15-s epochs across the whole night. These temporally ordered features are concatenated to form a whole-night sequence for each modality, preserving the temporal context of pathological events. To address dataset size limitations and class imbalance, data augmentation strategies are subsequently applied to generate diverse training samples. Each modality-specific feature sequence is then processed by the proposed MAF-SleepNet, which incorporates modality-specific attention modules to learn informative intra-modality patterns and a fusion mechanism to capture complementary inter-modality relationships. Finally, the fused representation is used to classify the input into one of six categories, comprising healthy subjects and five clinically significant sleep disorder classes. The following subsections provide a detailed description of each stage in the proposed methodology.

### 3.1. Datasets

Previous studies [19,20,38,39,40,41,42,43] conducted on automatic multi-class sleep disorder classification have typically relied on a single dataset, predominantly CAPSD. While such an approach facilitates method development and evaluation within a controlled setting, it inherently limits the generalizability of the resulting models due to dataset-specific characteristics such as patient demographics, recording protocols, and device configurations [6,21]. CAPSD [48,49] contains PSG recordings from 108 subjects, of which only four have SDB and five have CDH, specifically narcolepsy. Relying solely on such a dataset can lead to issues including class imbalance, limited disorder diversity, and overfitting to dataset-specific properties.

In this study, the initial classification target was based on the seven major categories of sleep disorders defined in ICSD-3 [10] plus a healthy class. Since PSG is not required for the diagnosis of insomnia disorders and CRSWDs, these categories were excluded. The remaining five PSG-requiring categories were retained: SBDs, CDHs, parasomnias, SMDs, and other PSG-diagnosable disorders. Within these categories, the selected disorders comprised OSA for SBDs; narcolepsy type 1, narcolepsy type 2, and hypersomnia unspecified for CDHs; RBD for parasomnias; PLMD for SMDs; and NFLE for the “other” category, which in ICSD-3 is classified under sleep-related medical and neurological disorders.

To ensure that each of these target categories was adequately represented, this study integrates PSG recordings from three publicly available datasets, namely CAPSD, the Nationwide Children’s Hospital Sleep DataBank (NCH-SDB) [48,51,52], and ANPHY-Sleep Dataset (ANPHY) [53], into a combined dataset. This integration provides a broader representation of sleep disorders, recording environments, and signal characteristics, thereby enhancing the robustness and potential applicability of the proposed method. Table 2 summarizes the subset of recordings from each dataset included in this study, along with their associated sleep disorder diagnoses. The characteristics of each dataset, as well as the subset of recordings utilized in this study, are described in Supplementary Section S1.

### 3.2. Preprocessing

**Figure 3:**
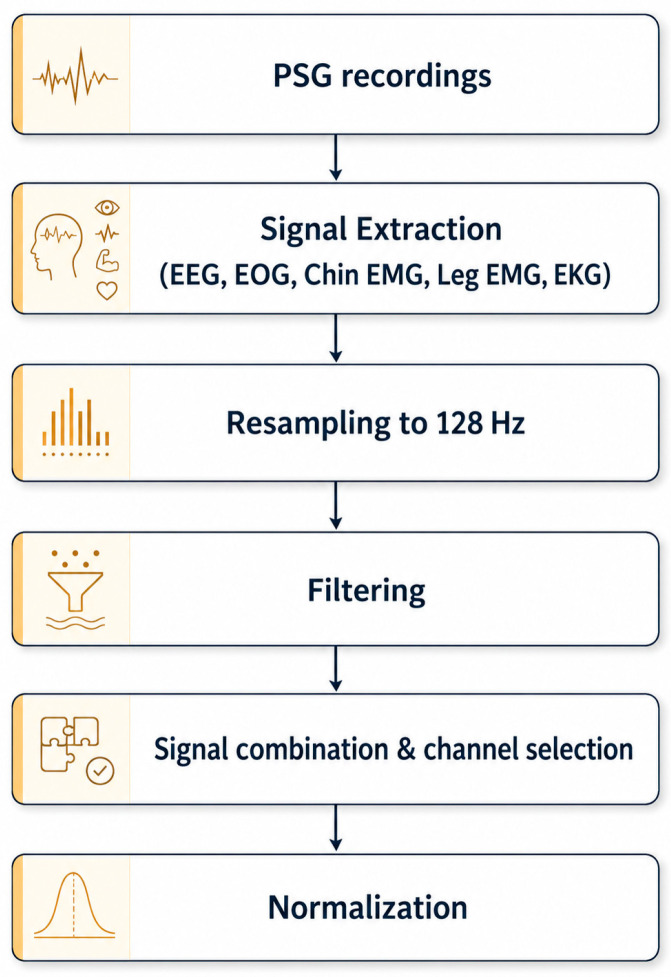
Overview of the preprocessing pipeline applied to the PSG signals.

This study aimed to classify sleep disorders using a multi-input DL model and to investigate the effect of a multimodal approach on model performance in order to identify the most effective signal modalities. For this purpose, five physiological signals commonly available in PSG recordings and present in all three datasets were utilized: EEG, EOG, leg EMG, chin EMG, and ECG. The overall preprocessing pipeline applied to these signals is illustrated in Figure 3, and the sequential steps are described in detail below.

***Step 1: Signal extraction***

In the first step, the five target physiological signals (EEG, EOG, leg EMG, chin EMG, and ECG) were extracted from each PSG recording.

***Step 2: Resampling***

As noted in Section 3.1, the sampling frequencies of the selected recordings varied between 100 Hz and 1000 Hz across datasets. To ensure uniform temporal resolution and enable joint processing across datasets, all extracted signal modalities were resampled to a common sampling rate of 128 Hz. Resampling was performed using MATLAB’s resample function, which combines interpolation with anti-aliasing filtering, thereby reducing spectral distortion during downsampling [54]. This strategy provided a standardized input representation for multimodal learning while balancing signal resolution and cross-dataset consistency.

***Step 3: Filtering***

Each resampled signal was bandpass-filtered according to the AASM guidelines [17,18], using the cut-off frequencies presented in Table 3, to remove baseline drift and high-frequency noise while preserving relevant physiological information.

***Step 4: Signal combination and channel selection:***

In some PSG recordings, the same physiological modality was measured using multiple channels. To obtain a consistent single-channel representation across all datasets, modality-specific combination rules were applied. For EOG, a bipolar derivation was computed by subtracting the left from the right ocular channel (EOG = ROC − LOC), enhancing horizontal eye movement detection and reducing noise. For chin EMG and ECG, bipolar signals were similarly derived (Chin EMG = Chin1 − Chin2; ECG = ECG1 − ECG2) to improve muscle activity isolation and cardiac waveform clarity, respectively. For leg EMG, the absolute values of the left and right channels were averaged (Leg EMG = (|LLEG_EMG| + |RLEG_EMG|) / 2) to represent overall leg activity. For EEG, the C4-A1 or C4-M1 channel was selected, depending on availability.

***Step 5: Normalization***

Finally, to standardize amplitude ranges and improve model convergence during training, z-score normalization was applied independently to each signal modality within each recording (i.e., per recording and per modality), using the mean and standard deviation computed from that recording’s own signal.

### 3.3. Whole-Night Epoch-Level Modality-Specific Feature Extraction

In previous studies on automatic sleep disorder classification, PSG recordings were typically segmented into epochs of 5 [42], 10 [19,43], or 30 [20,38,39,40] seconds, and the patient’s overall clinical diagnosis was assigned as the label for each epoch. This labeling strategy suffers from a fundamental limitation: a large proportion of epochs correspond to normal or non-pathological activity, yet they are still labeled with the disorder. As a result, the model is prone to learning subject-specific characteristics or dataset biases rather than capturing the true pathophysiological patterns of the disorder. Consequently, this approach not only introduces significant label noise but also reduces the ability of the model to generalize across patients and datasets.

To address this issue, our approach considers the entire overnight PSG recording as a single sequence, thereby preserving the temporal structure across the whole night. Nonetheless, directly using raw PSG signals was not feasible due to the huge data size. For instance, after resampling to 128 Hz, a 6–9 h PSG recording corresponds to approximately 2.76–4.15 million data points per channel, which poses significant challenges for both computational efficiency and model training stability. To overcome these limitations, each signal modality was segmented into consecutive 15-s epochs. From each epoch, modality-specific features were extracted, and the resulting features were concatenated in temporal order across the whole night. Importantly, segmentation into epochs was used only for feature extraction, whereas labeling was applied exclusively at the recording level, where a single clinical diagnosis was assigned to the entire overnight PSG sequence.

The 15-s window was chosen in preference to the conventional 30-s epoch, which is the AASM standard for manual sleep staging, where a single stage is assigned to each epoch, and is not intended for localizing episodic pathological events. Because many sleep-disorder events are considerably shorter than a staging epoch, a 15-s window doubles the temporal resolution of the whole-night feature sequence and localizes such events more precisely. This choice also balances temporal resolution against computational efficiency: shorter windows would lengthen the whole-night sequence and provide fewer samples per epoch, reducing the reliability of the spectral and complexity features, whereas the adopted window retains 1920 samples per epoch at 128 Hz—sufficient for stable feature estimation, including the low-frequency EEG bands. Moreover, because classification is performed at the recording level, each decision relies on aggregate patterns across the whole night rather than on isolated short events; brief sub-epoch transients, such as limb movements or K-complexes, therefore remain reflected in burst-sensitive extracted features even when their exact timing is not preserved.

This design is motivated not only by the lack of reliable epoch-level annotations in publicly available datasets [49,51,53] for many sleep disorders, but also by the inherent episodic nature of these conditions. Pathological events often occur intermittently and are confined to specific sleep stages or short temporal intervals [10,46,47], while large portions of the recording remain physiologically normal. Therefore, assigning the same diagnosis to every short epoch is not clinically meaningful and introduces artificial label noise.

By contrast, the proposed strategy preserves the temporal continuity of the full-night recording while avoiding misleading supervision at the epoch level, resulting in a more physiologically consistent and clinically valid representation for model learning. This design is also consistent with clinical diagnostic practice, where sleep disorders are defined at the patient level rather than at the short-epoch level.

The extracted features for each modality are summarized in Table 4. The feature set was not arbitrarily selected; rather, it was designed based on physiological relevance, prior literature, and commonly used signal descriptors in sleep analysis. Specifically, the selected features were chosen to capture complementary aspects of the signals, including spectral characteristics, amplitude variations, signal complexity, and temporal dynamics, which are known to reflect underlying neurophysiological and physiological processes associated with sleep disorders as reported in prior studies [6,21,55].

A consistent set of five features was derived from EOG, chin EMG, leg EMG, and ECG, whereas a broader set of eleven features was obtained from EEG. This reflects the central importance of EEG in sleep research and clinical practice, as many sleep disorders are primarily expressed through alterations in cortical oscillatory activity. Accordingly, EEG features were chosen to capture information across spectral bands (delta (δ, 0.5–4 Hz), theta (θ,4–8 Hz), alpha (α, 8–13 Hz), beta (β, 13–30 Hz)) [56], entropy-based measures of signal irregularity [55,57], and temporal complexity indices [20], providing a rich description of neural dynamics, which are closely linked to sleep stage transitions and pathological alterations in sleep disorders.

In contrast, peripheral modalities such as EMG, EOG, and ECG were represented with a more compact feature set of five descriptors each. For EMG (chin and leg), features emphasized muscle tone and phasic activity, which are essential for identifying phenomena such as REM sleep atonia and PLMDs. For EOG, features highlighted eye movement dynamics (e.g., RMS [58], line length [58], zero-crossing [57]), which are crucial for detecting REM-related activities. For ECG, the selected features were designed to capture autonomic signatures of sleep disturbances, including heart rate variability proxies, amplitude-based measures, and entropy [55]. No explicit feature selection or dimensionality reduction was applied, as the features were predefined based on physiological relevance and used in a temporal sequence format. Moreover, due to the episodic nature of sleep disorders, feature relevance is inherently time-dependent, making global feature selection less suitable.

The feature vectors extracted for each 15-s epoch from each modality can be expressed as:

(1) $$\begin{array}{c} F_{EEG}\in R^{11},\;\;\;\;\;\;\;\;\;\;F_{EOG}\in R^{5},\;{\;\;\;\;\;\;\;\;\;F}_{ChinEMG}\in R^{5},\\ \;F_{LegEMG}\in R^{5},\;\;\;\;\;\;\;\;\;F_{ECG}\in R^{5}\; \end{array}$$

Across the entire night, these epoch-level feature vectors were concatenated sequentially, yielding modality-specific sequences of length N, where N denotes the number of 15-s epochs in the recording (typically N≈1440−2160 for 6–9 h). Thus, the input representation for each subject can be described as:

(2) $$X_{EEG}\in R^{N\times 11},\;\;\;\;\;X_{EOG}\in R^{N\times 5},\;{\;\;\;\;X}_{ECG}\in R^{N\times 5}\;$$

(3) $$X_{ChinEMG}\in R^{N\times 5},\;{\;\;\;\;\;\;\;X}_{LegEMG}\in R^{N\times 5}$$

This multimodal feature extraction strategy ensures that both central and peripheral manifestations of sleep disorders are adequately represented in the model input. Detailed mathematical definitions of all features are provided in Supplementary Section S2.

### 3.4. Data Augmentation

MAF-SleepNet was designed as a feature-driven hybrid DL model trained from scratch, without relying on pre-trained feature extractors or transfer learning. Such models are inherently data-hungry and require large, diverse, and well-balanced datasets to achieve robust generalization [62,63]. However, the datasets employed in this study were relatively limited in size and exhibited class imbalance. Specifically, the combined dataset consisted of only 141 PSG recordings, distributed as 22 RBD, 37 NFLE, 15 CDH, 24 OSA, 20 PLMD, and 23 healthy controls. This scarcity of training samples, particularly within certain diagnostic categories, increases the risk of overfitting and reduces the ability of the model to generalize to unseen subjects [62,63]. To mitigate these limitations, we applied a two-step data augmentation strategy consisting of two operations applied synchronously across all modalities: (i) temporal shifting [64,65] and (ii) Gaussian noise injection [66,67].

First, each resampled PSG signal $s_{m}\left[n\right]\left(m\in \left\{EEG,EOG,ChinEMG,LegEMG,ECG\right\},f_{s}=128\;\mathrm{Hz}\right)$ was shifted at the raw signal level by integer offsets in $\Delta t\;\epsilon \;\left\{0,1,\ldots ,14\right\}$ seconds. This temporally shifted signal can be expressed as:

(4) $$s_{m}^{\Delta t}\left[n\right]=s_{m}^{\Delta t}\left[n+\Delta tf_{s}\right],\;\;\;\;\;n=1,2,\ldots ,\;T$$

where *T* denotes the total number of samples in the resampled signal. Each shifted signal was then segmented into non-overlapping 15-s epochs, and modality-specific features were extracted as described in Section 3.3.

Second, Gaussian noise [66,67] was added to the raw PSG signals to simulate realistic sensor-level perturbations and improve robustness to noise. The noise-augmented signal can be expressed as:

(5) $${\tilde{s}}_{m}\left[n\right]=s_{m}\left[n\right]+\epsilon \left[n\right],\;\;\;\;n=1,2,\ldots ,\;T$$

where $\epsilon \left[n\right]∼N\left(0,\;\sigma^{2}\right)$ represents zero-mean Gaussian noise with variance $\sigma^{2}$. In this study, the noise level was set to σ=0.01, ensuring that the perturbations were small enough to preserve physiological characteristics while still introducing variability. As with temporal shifting, each noise-augmented signal was segmented into non-overlapping 15-s epochs, and modality-specific features were extracted as described in Section 3.3.

As a result, each PSG recording yielded 15 augmented whole-night feature sequences from the raw signals and another 15 from the noise-perturbed signals, leading to a total of 30 augmented realizations per subject. The augmentation factor of 30 follows directly from the 15-s epoch structure: temporal shifting at a one-second resolution yields 15 distinct, non-redundant epoch alignments (0–14 s), which, when applied to both the original and the Gaussian-noise-perturbed signal, give 30 realizations per recording—the maximum that preserves signal fidelity without producing near-duplicate samples. Augmentation was therefore applied uniformly at this ceiling, while class imbalance was instead addressed separately during training-set construction (Section 4.1.1). Class-proportional augmentation was not adopted, since minority classes could not be augmented beyond this maximum without distorting the temporal alignment or generating near-duplicate variations of their few source subjects, which would increase the risk of overfitting under the strict subject-independent protocol. The input dimensions for each augmentation followed the same structure as in Section 3.3. An overview of the resulting augmented dataset, including the distribution of recordings and sequences per diagnostic category, is provided in Table 5. To ensure methodological integrity and prevent potential data leakage, augmented samples were handled in a subject-aware manner. By construction, each augmented realization is a deterministic, subject-local transformation of a single recording’s own raw signal; thus, no augmented sample is synthesized by combining data from different subjects and therefore cannot, by design, carry information about any other subject. Specifically, all augmented sequences derived from a given PSG recording were treated as belonging to the same subject and preserved as a single group for subsequent dataset partitioning. During a strictly subject-independent train–test split, these grouped samples were assigned exclusively to either the training or the test set, but never both. The same subject-level grouping strategy was consistently maintained during the subsequent subject-independent 4-fold cross-validation procedure. Moreover, all preprocessing and feature-extraction steps were performed strictly per recording and per modality. These properties jointly preclude any information leakage from the temporal-shifting and noise-augmentation steps.

### 3.5. Proposed MAF-SleepNet

**Figure 4:**
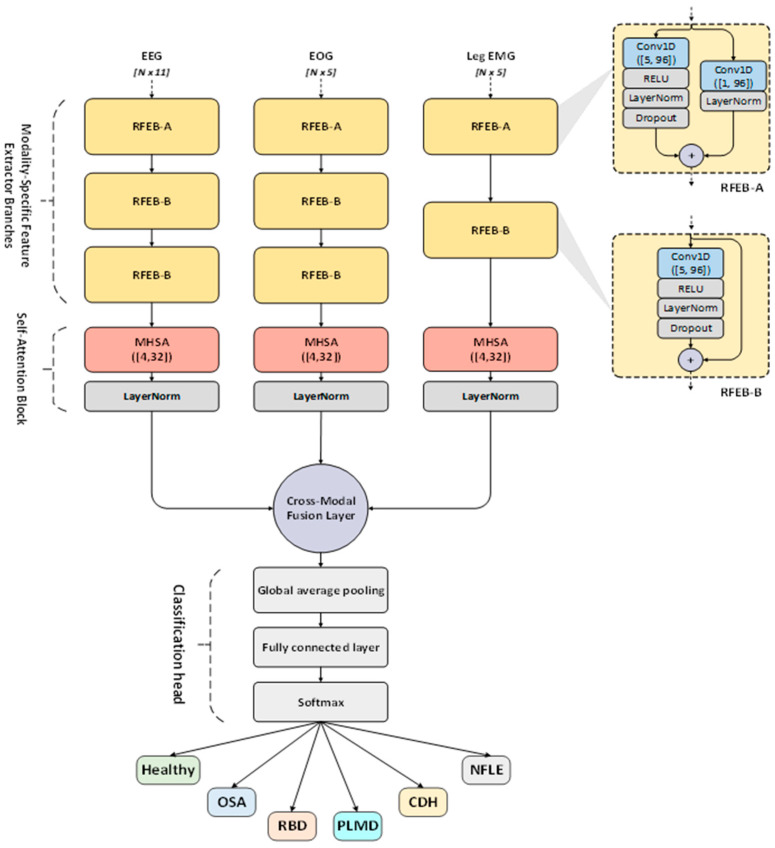
Detailed architecture of the proposed MAF-SleepNet for multi-class sleep disorder classification. The internal structures of the Residual Feature Extractor Blocks (RFEB-A and RFEB-B) are illustrated on the right. The notation “Conv1D ([k, c])” denotes a one-dimensional convolutional layer, where k represents the kernel size, and c indicates the number of output channels (filters). “MHSA ([4, 32])” denotes 4 attention heads and an embedding dimension of 32.

In this study, we developed a novel feature-driven hybrid DL model, named MAF-SleepNet, specifically designed for robust and accurate multi-class classification of sleep disorders by addressing the limitations and gaps of existing approaches. The proposed network integrates handcrafted feature extraction with DL-based representation learning, enabling effective modeling of temporal and cross-modal dependencies. To the best of our knowledge, MAF-SleepNet is the first architecture that simultaneously processes multiple physiological modalities from PSG recordings within a unified framework for this task. In addition, the model incorporates modality-specific attention mechanisms that selectively emphasize the most informative features, marking the first application of such mechanisms to this problem. Complementary information across modalities is further exploited through a cross-modal fusion layer, enabling a holistic representation of sleep pathophysiology. The final architecture of MAF-SleepNet was determined through extensive ablation studies, which systematically evaluated the effects of input modality combinations, the depth of residual feature extractor blocks, and the inclusion of attention mechanisms. These investigations guided the design of a model that not only learns rich intra-modality representations but also effectively integrates cross-modal dependencies, ultimately achieving state-of-the-art performance in multi-class sleep disorder classification. The detailed architecture of MAF-SleepNet is illustrated in Figure 4.

The overall architecture of MAF-SleepNet follows a structured, multi-level design that processes physiological signals in a modality-specific yet integrative manner. As illustrated in Figure 4, the input to the model consists of temporally ordered sequences of handcrafted features extracted from multiple PSG modalities, where EEG is represented by 11 features per epoch and EOG, chin EMG, leg EMG, and ECG are each represented by 5 features per epoch. Each modality-specific sequence is first processed through a dedicated Modality-Specific Feature Extractor Branch (MSFEB) using Residual Feature Extractor Blocks (RFEBs), which capture modality-dependent patterns while preserving essential information through skip connections. To further enhance discriminative power, a modality-specific attention mechanism is applied, allowing the model to prioritize the most informative features within each modality. The resulting representations are then concatenated through a cross-modal fusion layer, which integrates complementary information across modalities to form a unified feature representation. Finally, this fused representation is passed through a classification head that assigns each subject to one of six target categories, comprising healthy controls and five clinically relevant sleep disorder classes.

MAF-SleepNet takes as input three PSG modalities, defined as $M=\left\{X_{EEG},X_{EOG},X_{LegEMG}\right\}$. For each modality m∈M, the input is a temporally ordered sequence of per-epoch features:

(6) $$X^{\left(m\right)}=\left[x_{1}^{\left(m\right)},\;\ldots ,\;x_{N}^{\left(m\right)}\right]\;\in \;R^{N\times d_{m}}$$

where N denotes the number of non-overlapping 15-s epochs in an overnight recording, and the feature dimensionalities are $d_{EEG}=11,\;d_{EOG}=5,d_{LegEMG}=5$, as described earlier. Each $X^{\left(m\right)}$ is standardized per recording via z-score normalization to reduce inter-subject and inter-dataset amplitude variability. Since N is determined by the length of each overnight recording, it varies across subjects (approximately 1440–2160 epochs). These variable-length sequences are left-padded to a common length within each mini-batch, and the temporal global average pooling in the classification head then yields a fixed-size representation independent of N, ensuring comparability across subjects and a single recording-level prediction.

Each modality-specific sequence $X^{\left(m\right)}$ is first processed through a dedicated MSFEB. Within each MSFEB, feature learning is carried out by a cascade of RFEBs, designed to capture modality-dependent temporal dependencies while maintaining stable gradient flow through skip connections [68]. Two types of RFEBs are employed: RFEB-A, which includes an additional projection layer in the residual path to align feature dimensions, and RFEB-B, which uses an identity skip connection without projection. As illustrated in Figure 4, EEG and EOG branches consist of one RFEB-A followed by two RFEB-Bs, while the leg EMG branch employs one RFEB-A and one RFEB-B. This configuration, determined through ablation analysis, balances model depth and efficiency while preserving effective representation learning across modalities. The internal operation of an RFEB can be expressed as:

(7) $$Z_{j}=Dropout\left(LayerNorm\left(ReLU\left(Conv1D\left(Z_{j-1}^{\left(m\right)}\right)\right)\right)\right)\;+R\left(Z_{j-1}^{\left(m\right)}\right),\;$$

where $Z_{j-1}^{\left(m\right)}$ is the RFEB input, and $R\left(.\right)$ denotes the residual path. For the input of the RFEB-A, $Z_{j-1}^{\left(m\right)}$ corresponds to $X^{\left(m\right)}$, and $R\left(.\right)$ is a 1×1 Conv1D projection. Conv1D is a causal convolution layer (kernel size = 5, filters = 96) that extracts local temporal dependencies, followed by normalization and dropout (*p* = 0.4).

After feature extraction with RFEBs, each modality-specific representation is refined through a multi-head self-attention (MHSA) mechanism [69] to prioritize the most informative features. This mechanism assigns a weight to each temporal feature, allowing the model to adaptively focus on salient signal components within each modality. Formally, for the output sequence of the final RFEB-B, ${Z_{RFEB}}^{\left(m\right)}$, attention scores are computed as:

(8) $$\alpha =Softmax\left(\frac{QK^{T}}{\sqrt{d_{k}}}\right)$$

(9) $$\;Q={Z_{RFEB}}^{\left(m\right)}W_{Q}$$

(10) $$K={Z_{RFEB}}^{\left(m\right)}W_{K}$$

(11) $$V={Z_{RFEB}}^{\left(m\right)}W_{V}$$

where $W_{Q}{{,\;W}_{K},\;W}_{V}\in \;R^{d_{m}\times d_{k}}$ are learnable projection matrices. The modality-specific attended features are then obtained as:

(12) $$\;{\tilde{Z}}^{\left(m\right)}=\alpha V\;$$

Within MAF-SleepNet, self-attention is computed in parallel across four heads, each with a dimensionality of 32 ($d_{k}=32$). The outputs of all heads are concatenated and linearly projected, followed by layer normalization to yield the final modality-specific attended representation. This mechanism enables MAF-SleepNet to emphasize discriminative modality-specific patterns, thereby enhancing robustness against irrelevant or noisy features. No explicit positional encoding is applied within the self-attention module: the sequences are processed in temporal order, and the preceding convolutional RFEB layers already encode the local temporal arrangement of epochs, so positional information is inherently available to the attention mechanism.

After modality-specific refinement with MHSA, the attended representations ${\tilde{Z}}^{\left(m\right)}\in \;R^{N\times 96}$ from EEG, EOG, and leg EMG branches are concatenated to form a joint representation through the cross-modal fusion layer. This layer performs feature-level concatenation across modalities while preserving the temporal order of epochs:

(13) $$F_{fused}=\left[{\tilde{Z}}^{\left(EEG\right)}∥{\tilde{Z}}^{\left(EOG\right)}∥{\tilde{Z}}^{\left(LegEMG\right)}\right]\in \;R^{N\times 288}\;$$

In this study, simple feature-level concatenation is adopted as the fusion strategy. As demonstrated in Section 4, additional post-fusion processing (e.g., deeper layers or attention) did not improve performance and was therefore not included in the final architecture. By explicitly modeling this fusion step, MAF-SleepNet captures complementary relationships across central (EEG) and peripheral (EOG, EMG) modalities, enabling a holistic representation of sleep pathophysiology. Unlike prior works that treat modalities independently, this design emphasizes information fusion as a core mechanism, ensuring that cross-modal dependencies are jointly learned before final classification.

After cross-modal fusion, the unified feature representation $F_{fused}$ is passed to the classification head. Global average pooling (GAP) is first applied along the temporal dimension to summarize the sequence into a compact vector of length 288. Because the branch-level self-attention adaptively up-weights the most informative epochs prior to pooling, the global average pooling operates on attention-refined rather than uniformly averaged features, helping to preserve localized, disease-relevant information. This vector is then fed into a fully connected layer (FCL) followed by a softmax activation to produce the final probability distribution over six target classes (healthy, CDH, OSA, RBD, NFLE, and PLMD). The classification head can be expressed as follows:

(14) $$\hat{y}=Softmax\left(FCL\left(GAP\left(F_{fused}\right)\right)\right)\;$$

where $\hat{y}$ denotes the predicted probability distribution across classes.

## 4. Results

This section presents the experimental evaluation of the proposed MAF-SleepNet for multi-class sleep disorder classification. First, the experimental setup is described, including the training strategy, evaluation metrics, and implementation details. Next, the comprehensive classification performance of the proposed MAF-SleepNet under a subject-independent validation scheme is reported. Finally, the results of ablation studies are presented, providing a systematic evaluation of the contributions of input modalities, network architecture configurations, and the attention mechanism to the overall performance.

### 4.1. Experimental Setup

#### 4.1.1. Training Strategy

The training and evaluation of MAF-SleepNet were designed to ensure methodological consistency and reliability. For this purpose, all ablation studies were conducted using the augmented dataset under a hold-out scheme, where the dataset was partitioned into training and test subsets as summarized in Table 6. The training set was constructed to achieve an approximately balanced class distribution across disorders, ensuring comparable representation of each category during model development. A subject-independent strategy [70] was strictly applied such that data from the same subject never appeared in both subsets. While this subject-independent hold-out scheme was employed for the ablation experiments, the final evaluation of MAF-SleepNet was additionally performed using a 4-fold cross-validation scheme [71] to obtain a more reliable estimate of generalization performance. Although data augmentation was applied prior to dataset partitioning, a strict subject-independent grouping strategy was enforced during the train–test split. Consequently, no augmented samples originating from the same subject or recording were present in both subsets, thereby preventing data leakage.

Prior to the ablation studies, a base model consisting of three RFEBs (one RFEB-A and two RFEB-Bs), a cross-modal fusion layer, and a classification head was employed to determine the optimal training configuration. Two optimization algorithms, Adam and stochastic gradient descent with momentum (SGDM), were evaluated in combination with three learning rates (0.01, 0.001, and 0.0001). In addition, the number of convolutional filters within the RFEBs was tuned across five values (32, 64, 96, 128, and 160) to determine the most effective feature extraction capacity. For this search, a validation subset was drawn from the training data, while the test set was reserved exclusively for final evaluation. The configuration yielding the best validation performance was selected and subsequently adopted for all ablation studies as well as the final evaluation of MAF-SleepNet. The selected hyperparameters, along with other fixed settings such as batch size, kernel size, and loss function, are summarized in Table 7. Model training was performed for a maximum of 20 epochs, and an early stopping strategy was applied to mitigate overfitting: training was automatically terminated if the validation loss did not improve for five consecutive epochs.

#### 4.1.2. Evaluation Metrics

To comprehensively assess the performance of MAF-SleepNet, multiple standard evaluation metrics were employed. All evaluations were conducted on the held-out test subset unless otherwise specified. Overall classification performance was first quantified using accuracy, defined as the proportion of correctly classified samples across all classes. However, given the moderate class imbalance in the dataset, relying solely on accuracy may provide a biased estimate of model performance. Therefore, additional class-specific and macro-averaged metrics were reported, including precision (Pre), sensitivity (Sen), specificity (Spe), and the F1-score. In the multi-class setting, macro-averaged values of these metrics were computed by taking the unweighted mean of the metric across all classes, ensuring equal contribution of each disorder regardless of its prevalence [72]. For cross-validation of the proposed MAF-SleepNet, all metrics were reported as the mean and standard deviation across folds. In addition, 95% confidence intervals (CIs) were computed for the macro-averaged metrics and the overall accuracy using Student’s t-distribution (*n* = 4 folds). Furthermore, McNemar’s test with continuity correction [73] was used to determine whether the performance differences between competing model configurations were statistically significant, based on paired per-sample correctness outcomes on the same test set. Statistical significance was defined as p < 0.05.

#### 4.1.3. Implementation Details

All experiments were implemented in MATLAB R2024a using the Deep Learning Toolbox. Model training was carried out with the built-in “trainnet” function. The experiments were executed on a workstation equipped with an NVIDIA GeForce RTX 4090 GPU, a 13th -generation Intel Core i9-13900K CPU, and 128 GB of RAM, running Windows 11.

### 4.2. Performance of the Proposed MAF-SleepNet

The inputs and final architecture of the proposed MAF-SleepNet for multi-class sleep disorder classification were determined through a series of systematic ablation studies. Specifically, the five physiological signals commonly recorded in PSG, namely, EEG, ECG, EOG, leg EMG, and chin EMG, were first evaluated individually and in different combinations to assess their contribution to classification performance. Based on these findings, the best-performing input configurations were further optimized by adjusting the depth of the RFEBs within the modality-specific branches and after the fusion stage. Finally, self-attention blocks were incorporated at different levels (after each MSFEB, after the cross-modal fusion layer, and at both levels simultaneously) to examine their effect on the optimized architecture. The full results of these systematic ablation studies are presented in Section 4.3. Collectively, these ablation studies demonstrated that the combination of EEG, EOG, and leg EMG, integrated through MSFEBs and enhanced with branch-level self-attention, yielded the highest classification performance. Guided by these results, this configuration was adopted as the final architecture of MAF-SleepNet. The details of the resulting MAF-SleepNet are summarized in Table 8 together with its learnable parameters.

**Figure 5:**
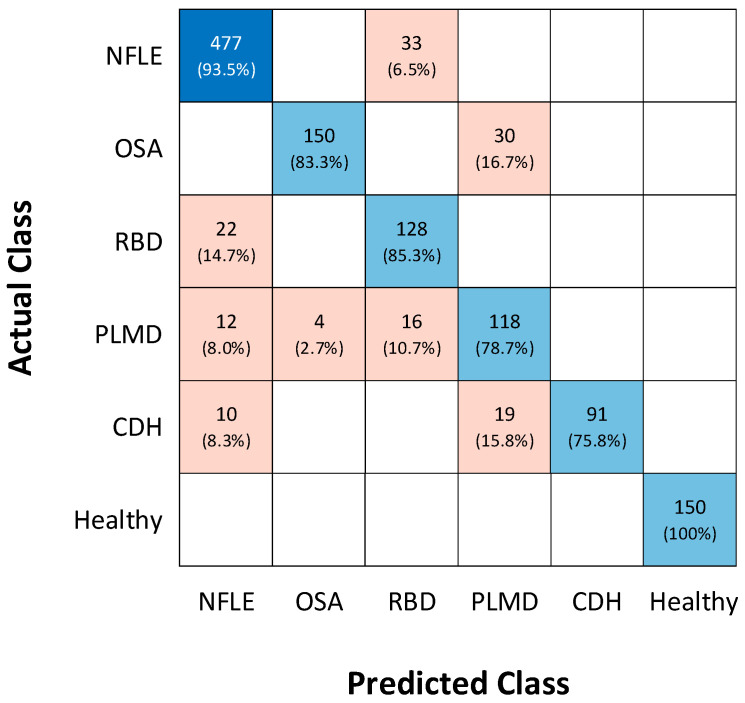
Confusion matrix of the proposed MAF-SleepNet under subject-independent evaluation.

The effectiveness of the final MAF-SleepNet architecture was first assessed under the subject-independent hold-out evaluation, which represents a strict scenario where no subject overlap exists between the training and test sets. The detailed per-class and averaged results are presented in Table 9. The model achieved an overall accuracy of 88.41%, with macro-averaged sensitivity, specificity, precision, and F1-score of 86.12%, 97.49%, 88.66%, and 86.89%, respectively. These results indicate that the network maintains balanced performance across disorders despite the heterogeneity of their pathophysiological signatures. Among the individual categories, NFLE reached the highest sensitivity (93.53%), reflecting the strong cortical signatures accessible from EEG. In comparison, OSA demonstrated outstanding specificity (99.63%) and precision (97.40%) but comparatively lower sensitivity (83.33%), suggesting a tendency to miss some positive cases. Disorders with overlapping motor manifestations, such as RBD and PLMD, exhibited relatively modest F1-scores (78.29% and 74.45%), consistent with the difficulty of discriminating subtle motor-related abnormalities. In contrast, CDH achieved perfect precision and specificity but limited sensitivity, likely due to the smaller number of positive samples. Finally, the healthy class was detected with perfect scores across all metrics, reflecting its distinct separation from pathological categories. Collectively, these findings demonstrate that while MAF-SleepNet performs robustly across diverse conditions, movement-related disorders remain the most challenging to classify. These observations are further supported by the confusion matrix presented in Figure 5, which provides a detailed view of class-wise predictions and misclassification patterns. In particular, notable confusion is observed between NFLE and RBD, between OSA and PLMD, and for PLMD and CDH with neighboring clinical categories, whereas the healthy class is separated almost perfectly.

To obtain a more reliable estimate of generalization performance, the final model was further evaluated under a subject-independent 4-fold cross-validation scheme. During this evaluation, strict subject-level separation was preserved within each fold. No original or augmented sample from the same subject/recording was ever present in either the training or validation subsets within any fold. The detailed per-class results are summarized in Table 10. MAF-SleepNet achieved an overall accuracy of 86.07 ± 3.66% (95% CI: 80.25–91.89), with macro-averaged sensitivity, specificity, precision, and F1-score of 82.79 ± 5.18%, 97.23 ± 0.75%, 83.66 ± 4.06%, and 82.67 ± 4.46% (95% CI: 75.57–89.77), respectively. Compared with the hold-out results, these findings confirm the robustness of the model while also highlighting variations across folds, particularly for disorders with fewer samples. For example, NFLE was detected with consistently high performance (96.27 ± 1.21% macro-F1), whereas conditions such as OSA and PLMD exhibited lower sensitivity (63.34 ± 2.61% and 68.00 ± 6.62%, respectively), indicating that motor and respiratory disorders remain more challenging to identify reliably. Moreover, the relatively large standard deviations observed for RBD and CDH indicate less consistent performance across folds, which may be attributed to the limited number of samples and the variability of disorder-specific patterns. Despite these challenges, the strong macro-specificity (97.23 ± 0.75%) demonstrates that false positives were kept at a minimum, underscoring the discriminative capacity of the proposed MAF-SleepNet.

For completeness, we also conducted a subject-dependent 4-fold cross-validation, where recordings from the same subject could appear in both the training and test sets. The results, presented in Table 11, show near-ceiling performance with an overall accuracy of 98.96 ± 0.66% (95% CI: 97.91–100.0) and macro-averaged sensitivity, specificity, precision, and F1-score all exceeding 98%. While these findings highlight the strong representational capacity of MAF-SleepNet, they should be interpreted with caution, as the subject-dependent setting does not fully reflect real-world clinical scenarios and may artificially inflate performance due to subject overlap. Nevertheless, this analysis provides an upper-bound estimate of the model’s capacity. It confirms that the proposed architecture is capable of learning highly discriminative features when inter-subject generalization constraints are relaxed.

### 4.3. Ablation Studies

In this study, the final input configuration and architecture of MAF-SleepNet were determined through a series of systematic ablation experiments, which are presented in this section. Specifically, Section 4.3.1 investigates the effect of individual and combined input modalities on the performance of a base model consisting of three RFEBs (one RFEB-A and two RFEB-Bs), a cross-modal fusion layer, and a classification head. Section 4.3.2 evaluates the impact of network configuration by varying the number of RFEBs within the MSFEB branches and after the cross-modal fusion layer. Finally, Section 4.3.3 examines the contribution of attention mechanisms by incorporating MHSA modules after each MSFEB, after the fusion layer, and at both locations simultaneously. Together, these experiments provide a comprehensive understanding of the design choices that drive the effectiveness of MAF-SleepNet.

#### 4.3.1. Impact of Input Modalities

Sleep disorders are heterogeneous in nature, with distinct pathophysiological signatures observable across multiple physiological domains. EEG is the primary modality for sleep disorder detection, as it captures cortical dynamics underlying arousals, stage-dependent abnormalities [17,23], and epileptiform events such as those observed in NFLE [74,75]. EOG provides key information on REM-related disturbances, particularly RBD [46,47], while EMG is indispensable for detecting motor phenomena such as PLMD and REM atonia [17,23,76]. In contrast, ECG primarily reflects autonomic correlates of sleep fragmentation and cardiorespiratory disturbances, most notably in OSA, but offers limited disorder-specific information when used in isolation [45,77,78,79]. Relying on a single modality risk overlooking these complementary sources of information, which motivates the design of a multi-input, multi-modal classification framework. From the perspective of information fusion, integrating central, ocular, muscular, and autonomic modalities has the potential to substantially enhance discriminative power and improve the robustness of automatic sleep disorder classification.

Guided by these considerations, we first systematically evaluated the discriminative capacity of each individual modality and its combinations using the base model, which consisted of one RFEB-A and two RFEB-Bs in each MSFEB, followed by a cross-modal fusion layer (omitted for single-modality inputs) and a classification head. Table 12 summarizes the results obtained across single-, double-, triple-, quadruple-, and all-modality configurations in terms of accuracy and macro-F1, along with the number of trainable parameters. To ensure the reliability of the findings, each experiment was conducted twice, and the average performance values are reported. Among the single-modality inputs, EEG achieved the highest performance (71.19% accuracy, 67.17% macro-F1), reflecting its indispensable role in characterizing sleep and detecting abnormalities related to various sleep disorders. EOG and both EMG (chin and leg EMG) also provided competitive results, while ECG showed limited discriminative ability when used alone (47.18% accuracy, 36.74% macro-F1).

To further investigate the limited performance of ECG, a permutation-based feature importance analysis was conducted on the ECG-only configuration (S2) using the test set. In this analysis, each feature was randomly permuted across samples to quantify its impact on model performance. The results, presented in Table 13, indicate that all extracted ECG features contributed to classification performance, as evidenced by consistent reductions in macro-F1 when individually perturbed. The observed macro-F1 drops ranged from 0.0943 to 0.1374, with peak-to-peak amplitude and line length producing the largest decreases. Despite these contributions, the ECG-only configuration remained substantially weaker than other single-modality inputs. These findings indicate that while ECG contains informative signals, its overall contribution remained limited in the present multi-class setting.

Combining modalities led to substantial improvements, underscoring the advantages of information fusion in multi-class sleep disorder classification. By integrating complementary physiological signals, the model was able to exploit cross-modal dependencies that are not accessible through single inputs. Notably, the triple-modality configurations T1 (EEG + EOG + Leg EMG) and T6 (EEG + Leg EMG + Chin EMG) achieved the best results, with accuracies above 81% and macro-F1 scores close to 79%. In particular, according to McNemar’s test, the two best triple-modality configurations significantly outperformed the strongest single-modality model, namely the EEG-only model: T1 ($\chi^{2}\left(1\right)=45.28$, p < 0.001; b = 201, c = 86) and T6 ($\chi^{2}\left(1\right)=97.66$, p <0.001; b = 201, c = 45). These findings provide statistical evidence that multimodal fusion offers a significant advantage over single-modality input. The four-modality configuration Q4 (EEG + EOG + Leg EMG + Chin EMG) also performed competitively (80.24% accuracy, 77.10% macro-F1). Interestingly, incorporating all five modalities simultaneously did not yield further gains and, in fact, degraded performance compared to the optimal subsets. This outcome suggests that indiscriminate fusion may introduce redundant or weakly informative signals and increase the risk of overfitting due to the increased feature dimensionality. These findings highlight that the effectiveness of information fusion depends not only on the number of modalities but also on their complementarity and relevance to the disorders under study. Based on these observations, T1, T6, and Q4 were selected as representative input modality configurations for subsequent ablation experiments.

#### 4.3.2. Optimization of Model Configuration

The architectural configuration of deep networks strongly influences their ability to capture discriminative features while maintaining generalization. In the context of MAF-SleepNet, this primarily concerns the number of RFEBs employed within the modality-specific branches of the MSFEB and after the cross-modal fusion layer. Insufficient depth may limit the extraction of complex intra-modality patterns, whereas excessive depth can lead to overfitting and unnecessary computational overhead. Building on the results of Section 4.3.1, where T1 (EEG + EOG + Leg EMG), T6 (EEG + Leg EMG + Chin EMG), and Q4 (EEG + EOG + Leg EMG + Chin EMG) were identified as the most effective input configurations, we systematically examined how variations in network depth affect performance. Two structural factors were considered: (i) the number of RFEBs assigned to each input modality branch (branch depth) and (ii) the number of RFEBs appended after the cross-modal fusion layer (fusion depth). These experiments were designed to identify the optimal representational capacity of the network while avoiding redundancy and overfitting. All experiments were repeated twice, and average performance values are reported to ensure reliability.

**Figure 6:**
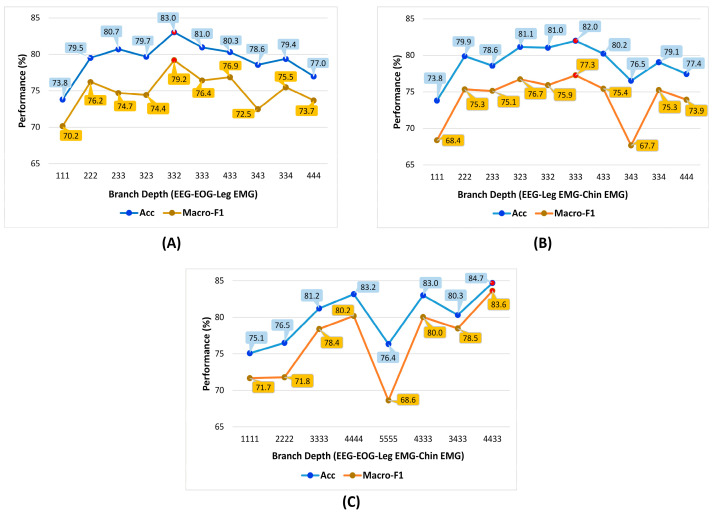
Effect of MSFEB branch depth on performance across the three best input configurations: (**A**) T1 (EEG + EOG + Leg EMG), (**B**) T6 (EEG + Leg EMG + Chin EMG), and (**C**) Q4 (EEG + EOG + Leg EMG + Chin EMG). Accuracy (blue) and macro-F1 (orange) are reported. Depth setting i-j-k (or i-j-k-l for Q4) denotes the number of RFEBs in each modality branch. The highest-performing configurations are highlighted with red circles.

Figure 6 illustrates the effect of varying MSFEB branch depth on classification performance across the three best input configurations (T1, T6, and Q4). For all cases, shallow settings (e.g., depth = 111) yielded relatively low accuracy and macro-F1 scores, indicating an insufficient representational capacity. Increasing the number of RFEBs improved performance, with each configuration achieving its best results at moderate depths: 332 for T1 (83.02% accuracy, 79.18% macro-F1), 333 for T6 (81.99% accuracy, 77.27% macro-F1), and 4433 for Q4 (84.71% accuracy, 83.63% macro-F1). Beyond these points, further increases in branch depth failed to yield additional gains and, in some cases, reduced performance, suggesting redundancy and overfitting. These findings underscore the need to carefully balance depth across modality branches to maximize discriminative power while preserving generalization.

**Figure 7:**
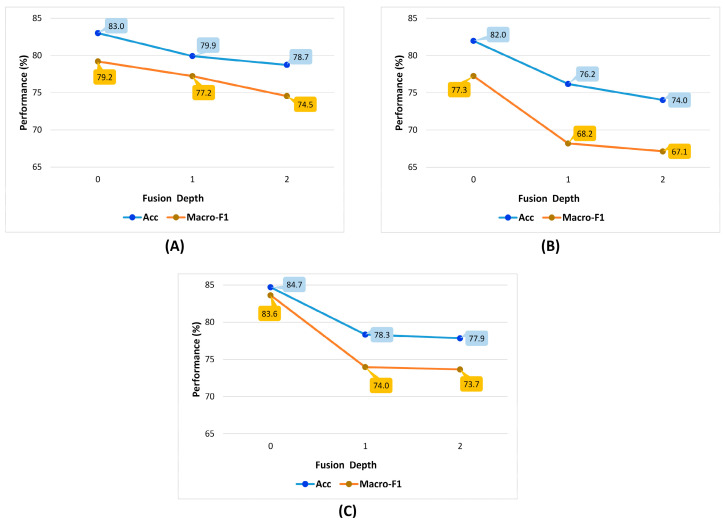
Effect of fusion depth on classification performance across the three best input configurations: (**A**) T1 (EEG + EOG + Leg EMG), (**B**) T6 (EEG + Leg EMG + Chin EMG), and (**C**) Q4 (EEG + EOG + Leg EMG + Chin EMG). Accuracy (blue) and macro-F1 (orange) are reported. “Fusion depth” denotes the number of RFEBs appended after the cross-modal fusion layer.

After fixing the branch depth at the best-performing settings for each configuration, we next examined the impact of increasing network depth after the cross-modal fusion stage. Figure 7 shows the effect of varying fusion depth, defined as the number of RFEBs appended after the fusion layer, across the three best input configurations. In all cases, the best performance was achieved when no additional RFEBs were included (fusion depth = 0). Adding one or more RFEBs consistently reduced both accuracy and macro-F1, with declines of up to 4–5% depending on the configuration. This outcome suggests that once modality-specific features are integrated at the fusion stage, further convolutional processing introduces redundancy and overfitting rather than improving discriminative capacity. These findings indicate that the fusion layer alone is sufficient for modeling cross-modal dependencies and that model complexity should be concentrated within the modality-specific branches rather than after fusion.

#### 4.3.3. Impact of Attention Mechanisms

While convolutional layers in MAF-SleepNet effectively capture local temporal dependencies within each modality, they may be limited in modeling long-range interactions and selectively prioritizing the most informative features. Attention mechanisms, particularly MHSA [69], provide a principled way to address these limitations by dynamically weighting temporal features and emphasizing salient modality-specific or cross-modal patterns. Building on the results of Section 4.3.1 and Section 4.3.2 (where T1, T6, and Q4 were identified as the most effective input configurations and their optimal architectural depths were determined), we next investigated the role of attention in further enhancing performance. For this purpose, we integrated self-attention blocks, each consisting of an MHSA module followed by layer normalization, at three levels: (i) after each modality-specific branch (branch-level attention), (ii) after the cross-modal fusion layer (fusion-level attention), and (iii) at both levels simultaneously (combined attention). These experiments were designed to determine whether attention provides complementary benefits beyond convolutional feature extraction and to identify the most effective placement strategy. The findings from this analysis establish the role of attention in shaping the final architecture of MAF-SleepNet.

Table 14 summarizes the impact of different attention strategies across the three best-performing input configurations (T1, T6, and Q4), together with the corresponding number of trainable parameters. The results show that the benefit of attention is not uniform, but rather depends on the characteristics of the input modalities and the stage at which attention is applied. For the T1 configuration (EEG + EOG + Leg EMG), branch-level attention produced a marked performance improvement, increasing accuracy from 83.02% to 88.41% and macro-F1 from 79.18% to 86.89%. This improvement was statistically significant (McNemar’s test, $\chi^{2}\left(1\right)=35.07$, p < 0.001; 98 vs. 30 discordant predictions in favor of the attention-based model). This indicates that selectively weighting modality-specific features before fusion is particularly effective when heterogeneous modalities capturing complementary central, ocular, and muscular dynamics are combined. In contrast, fusion-level attention substantially degraded performance, suggesting that post-fusion attention disrupted rather than enhanced the integrated representation. Combined attention partially mitigated this effect but still performed worse than branch-level attention, underscoring that additional complexity does not necessarily translate into better generalization.

In the case of T6 (EEG + Leg EMG + Chin EMG), the highest performance was observed with fusion-level attention, achieving 85.79% accuracy and 83.41% macro-F1. This configuration appears to benefit from emphasizing cross-modal dependencies at the integration stage, where EEG and motor-related activity jointly capture disorder-specific patterns, particularly for conditions such as PLMD. Branch-level attention offered only modest gains over the baseline, while combined attention again reduced performance. By contrast, for Q4 (EEG + EOG + Leg EMG + Chin EMG), the best performance was achieved without attention, reaching 84.71% accuracy and 83.63% macro-F1. The diversity of four complementary modalities in this case already provides a sufficiently rich and discriminative feature space such that introducing additional attention mechanisms mainly increased network complexity and led to overfitting rather than improved generalization. These results highlight that attention is not universally beneficial; its effectiveness depends on the composition of input modalities and the level at which it is applied. This analysis ultimately informed the design of the final MAF-SleepNet architecture, where attention mechanisms are integrated selectively to maximize their utility. Among the evaluated settings, T1 (EEG + EOG + Leg EMG) with branch-level self-attention not only achieved the highest overall performance but also offered a favorable balance between accuracy, generalization, and model complexity. Therefore, this configuration was adopted as the foundation of the proposed MAF-SleepNet architecture. Finally, two additional analyses were conducted on this final configuration to assess our data-level design choices. First, to isolate the contribution of data augmentation, the model was trained using only the original, non-augmented recordings under the identical subject-independent hold-out protocol; without augmentation, accuracy and macro-F1 dropped to 78.57% and 76.11%, compared with 88.41% and 86.89% obtained with the full 30× augmentation, confirming that augmentation is essential given the limited cohort of 141 recordings. Second, replacing the standard cross-entropy loss with class-weighted cross-entropy or focal loss (γ = 2) [80] did not improve performance (macro-F1 of 83.31% and 78.22%, respectively, versus 86.89% for the default), indicating that the approximately balanced training-set construction already handles the class imbalance effectively and that additional loss-level rebalancing over-corrects the objective.

## 5. Discussion

This study aimed to develop a high-performance DL model for the classification of multiple sleep disorders using PSG recordings. To this end, we proposed MAF-SleepNet, a novel multi-modal attention-enhanced fusion network that preserves the temporal continuity of whole-night recordings while integrating complementary information from EEG, EOG, and leg EMG signals. Experiments were conducted on a comprehensive dataset created by combining three publicly available PSG databases. Experimental results demonstrated that MAF-SleepNet achieved 88.41% accuracy and a macro-F1 score of 86.89% under a subject-independent hold-out evaluation. In addition, subject-independent four-fold cross-validation resulted in an accuracy of 86.07 ± 3.66% and a macro-F1 score of 82.67 ± 4.46%, while subject-dependent four-fold cross-validation yielded near-ceiling performance (98.96 ± 0.66% accuracy and 98.93 ± 0.60% macro-F1), which should be interpreted with caution due to potential subject-specific bias. While the model demonstrates strong performance across different evaluation protocols, the subject-independent results should be considered the primary and clinically meaningful indicator of generalization performance. In addition, the proposed MAF-SleepNet model has a relatively low number of trainable parameters (293.4 K), indicating its computational efficiency and potential suitability for practical and clinical deployment.

Most existing studies on automatic sleep disorder classification have focused on a single disorder, with the majority targeting OSA due to its high prevalence and clinical relevance [6,21,50]. Although these single-disorder approaches have achieved high accuracy, they are inherently limited in practice, as clinicians routinely encounter a wide variety of sleep disorders. To overcome this limitation, a limited number of recent studies [19,20,38,39,40,41,42,43] have attempted to address multi-class classification; however, these efforts still exhibit several important shortcomings. In particular, existing multi-class studies have relied on a single dataset [19,20,38,39,40,41,42,43], most frequently the CAPSD [19,20,38,39,40,42,43], which restricts disorder diversity and hampers generalizability. Moreover, the ICSD-3 framework has rarely been systematically considered. In some cases, studies have included disorders that do not require PSG for diagnosis, such as insomnia [19,20,38,39,40,42,43], whereas others have omitted PSG-requiring conditions such as PLMD [38] or NFLE [38,43]. Consequently, the diagnostic scope of existing multi-class studies does not fully align with clinically meaningful categories. In contrast, the present study explicitly adopted the ICSD-3 taxonomy [10] as the basis for class selection, focusing exclusively on five PSG-requiring categories: OSA from SBDs, RBD from parasomnias, PLMD from SMDs, narcolepsy and hypersomnia from CDHs, and NFLE for the remaining conditions. Furthermore, by integrating recordings from three distinct datasets (CAPSD [48,49], NCH-SDB [48,51,52], and ANPHY [53]), we constructed a more heterogeneous and representative dataset, thereby enhancing disorder coverage and improving the robustness and generalizability of the proposed framework. Importantly, the inclusion of NCH-SDB, which consists of pediatric PSG recordings, further broadened the demographic spectrum of the dataset and increased the potential applicability of the model to a wider population, offering a more realistic foundation for clinical translation.

A major methodological limitation in most existing multi-class sleep disorder studies concerns the labeling strategy employed. In seven of the eight published works [19,20,38,39,40,42,43], PSG recordings were divided into short epochs, with each epoch subsequently assigned the patient’s overall clinical diagnosis as its label. Such an approach overlooks the inherently episodic nature of sleep disorders, which typically manifest only during specific sleep stages or restricted temporal intervals rather than throughout the entire night. For instance, abnormal respiratory events in OSA arise intermittently [17,44,81], RBD-related behaviors occur exclusively during REM sleep [46,47], and PLMD is characterized by discrete periodic limb movements [10,17,23]. As a result, large proportions of physiologically normal epochs are mislabeled as pathological, thereby introducing substantial label noise. This not only obscures the true temporal distribution of disorder-specific events but also biases models toward learning subject-specific signal characteristics instead of genuine pathological patterns, an effect that becomes particularly pronounced when evaluations are conducted under subject-dependent protocols, ultimately inflating reported performance and undermining clinical validity, and therefore the results should be interpreted with caution. In the present study, this issue was addressed by analyzing entire overnight PSG recordings as temporally ordered sequences, thereby preserving the episodic manifestation of sleep disorders and avoiding the artificial mislabeling of normal epochs. Consequently, the results presented here are not directly comparable with those of earlier studies, owing to these fundamental methodological differences. Nevertheless, if a comparison is to be drawn, the subject-dependent evaluation results obtained in this study, which achieved near-ceiling performance, may be regarded as more analogous to previously reported findings, although subject-independent outcomes remain the more clinically relevant benchmark.

Another important methodological concern in prior studies relates to the evaluation protocol. In many multi-class classification works [19,20,38,39,40,42,43], subject-independent data partitioning was not enforced, allowing epochs from the same patient to appear simultaneously in both training and test sets. This practice leads to data leakage, whereby models exploit subject-specific signal characteristics rather than learning disorder-related patterns, ultimately resulting in overly optimistic performance estimates. Such inflated metrics substantially limit the clinical credibility of these approaches, as they do not reflect the generalization ability required in real-world diagnostic scenarios. In contrast, the present study rigorously employed a strictly subject-independent evaluation protocol across all experiments, thereby ensuring that no data from the same individual was used in both training and testing. This strategy provided a more realistic assessment of model performance and better reflects clinical applicability. For completeness and comparability with earlier work, subject-dependent results were also reported, yielding near-ceiling scores; however, the subject-independent findings constitute the most reliable benchmark for evaluating the true diagnostic potential of the proposed framework.

To further contextualize the performance of the proposed model, a comparison with representative studies on the CAPSD dataset, along with additional evaluation settings of the proposed framework, is summarized in Table 15. Under a subject-dependent evaluation on CAPSD, MAF-SleepNet achieves near-ceiling performance (macro-F1 ≈ 99.50), which outperforms most previously reported multi-class studies [20,38,39,40,42,43] and achieves a modest improvement of approximately 0.5% in macro-F1 compared to the best-performing study [19] under comparable evaluation conditions. In addition, when evaluated on the combined dataset using a subject-dependent protocol, the model maintains similarly high performance, further demonstrating its robustness across heterogeneous data sources. However, it is important to emphasize that most prior studies rely on subject-dependent and epoch-level evaluation strategies, which are known to introduce optimistic bias due to subject overlap and labeling assumptions.

In contrast, the subject-independent evaluation adopted in this study provides a more realistic and clinically meaningful assessment by ensuring that data from the same subject do not appear in both training and testing sets. Furthermore, unlike prior work, the proposed framework operates on whole-night recordings, thereby preserving the temporal structure of PSG signals and reducing the artificial inflation of performance associated with epoch-level labeling. Therefore, although direct numerical comparisons should be interpreted with caution, the proposed model demonstrates competitive performance under comparable settings while offering a more robust and clinically relevant evaluation through subject-independent and whole-night learning strategies.

To further contextualize the effectiveness of the proposed architecture, MAF-SleepNet was additionally compared against three modern DL baselines, namely a 1D-CNN, a CNN-BiLSTM, and a Transformer encoder, under an identical experimental setting. To ensure a fair comparison, all baselines were trained and evaluated in the same way as the proposed model, using the same T1 input (EEG + EOG + Leg EMG) and the same subject-independent train/test partition. Each baseline processes every modality through its own dedicated branch, whose outputs are then concatenated, as in MAF-SleepNet, and passed to a classification head consisting of a GAP layer, an FCL, and a softmax classifier. Only the per-branch feature extractor differs, thereby isolating the contribution of the RFEBs and modality-specific attention of MAF-SleepNet. The architectural and training details of the baselines are provided in Supplementary Section S3.

The classification results of all models, together with the corresponding McNemar’s test outcomes, are summarized in Table 16. MAF-SleepNet outperformed all three baselines by a substantial margin, achieving 88.41% accuracy and 86.89% macro-F1, compared with 78.02% and 73.56% for the 1D-CNN, 83.02% and 76.75% for the CNN-BiLSTM, and 70.44% and 67.84% for the Transformer. According to McNemar’s test on the paired per-sample predictions, all of these differences were statistically significant (all *p* < 0.001). Importantly, MAF-SleepNet attained this improvement with a parameter budget comparable to that of the 1D-CNN (293.4 K versus 290.8 K), indicating that these gains arise from its RFEBs and modality-specific attention rather than from an increased number of parameters.

### 5.1. Information Fusion

Most existing studies on automatic sleep disorder classification have relied on a single physiological modality, most frequently EEG [39,40,41] and, less often, ECG [38,43]. While such unimodal approaches can capture disorder-specific features within a limited domain, they fail to reflect the inherently multidimensional nature of sleep disorders, which manifest through diverse neural, muscular, ocular, and autonomic mechanisms. A smaller number of studies [19,20,42] have attempted to incorporate multiple PSG signals; however, these typically employ simple feature concatenation or train separate models for each modality with outputs combined at the decision level. Such strategies provide only limited integration and overlook the complex interdependencies across modalities. Consequently, both unimodal and loosely coupled multimodal approaches remain insufficient for robust multi-class classification and clinically reliable generalization.

In contrast to these limitations, the present study introduced MAF-SleepNet, a unified feature-driven hybrid architecture that explicitly models both intra- and inter-modality relationships. Each modality is first processed through a dedicated branch, with residual feature extraction and attention mechanisms, enabling the network to focus on the most informative temporal patterns within EEG, EOG, and leg EMG signals. The resulting modality-specific representations, already refined by residual feature extraction and modality-specific attention, are then integrated through a fusion layer that preserves complementary information and captures cross-modal dependencies. Applying this simple fusion to attention-refined rather than raw modality features allows the model to learn richer and more clinically meaningful representations of sleep disorder. Importantly, ablation studies confirmed the clear performance advantage of information fusion: while single-modality configurations such as EEG alone achieved around 71% accuracy and 67% macro-F1, multimodal combinations substantially improved performance, with the EEG + EOG + leg EMG configuration reaching 88.41% accuracy and 86.89% macro-F1 under subject-independent evaluation. Furthermore, the results revealed that not all modalities contribute equally: the selective integration of complementary modalities enhanced generalization, whereas the indiscriminate inclusion of additional signals such as ECG or chin EMG introduced redundancy and reduced performance. These findings highlight both the necessity of information fusion and the importance of modality selection, underscoring the advantages of an adaptive, attention-enhanced multimodal framework for reliable multi-class sleep disorder classification. Ultimately, this adaptive multimodal integration mirrors clinical practice, where PSG interpretation inherently relies on synthesizing evidence across multiple physiological domains.

### 5.2. Clinical Implications

The proposed MAF-SleepNet demonstrates several clinically relevant strengths and limitations that reflect the underlying pathophysiology of different sleep disorders. The model achieved particularly strong performance for NFLE and CDHs, with F1-scores of 92.53% and 86.26% under hold-out evaluation, and 96.27% and 75.88% under cross-validation, respectively. These results indicate that cortical abnormalities and hypersomnolence-related patterns are captured reliably by multimodal fusion, making automated screening for these conditions a promising clinical application. Performance for RBD was moderate (F1 = 78.29% hold-out; 84.83% cross-validation), suggesting that REM-related abnormal motor activity can be identified reasonably well, though occasional misclassifications remain. In contrast, OSA and PLMD exhibited relatively weaker performance, with F1-scores of 89.82% and 74.45% in hold-out evaluation and dropping to 73.64% and 66.50% in cross-validation. These findings highlight the inherent difficulty of capturing episodic respiratory and motor abnormalities, which are highly variable across patients and often confounded by signal artifacts. Notably, healthy subjects were distinguished with near-perfect accuracy (F1 = 100% hold-out; 98.91% cross-validation), demonstrating the framework’s ability to minimize false positives and reduce unnecessary diagnostic procedures.

A notable observation from the ablation experiments is that ECG, although expected to improve performance for OSA due to its sensitivity to cardiorespiratory disturbances, did not provide a meaningful contribution. To further examine this behavior, a permutation-based feature importance analysis was conducted on the ECG-only configuration of the base model. While all extracted ECG features contributed to classification performance to some extent, the overall performance of this configuration remained substantially lower than other modalities. A more detailed class-specific analysis, presented in Table 17, reveals that the contribution of ECG features is not uniform. In particular, features such as peak-to-peak amplitude, RR interval variability, and especially Shannon entropy exhibit a noticeable impact on OSA detection, as reflected by reductions in recall when perturbed. In contrast, other features, such as variance and line length, show negligible or inconsistent contributions. These observations indicate that ECG captures certain disorder-specific autonomic patterns, particularly for OSA, but does not provide a consistently informative representation across all features or classes.

Taken together, these findings suggest that the contribution of ECG is heterogeneous and not uniformly transferable across different sleep disorder categories. This helps explain why the inclusion of ECG did not lead to performance improvements in the multi-modal setting. Furthermore, it indicates that the limitation is likely related to the expressiveness of the extracted ECG features rather than the absence of relevant physiological information. Therefore, more advanced ECG representations or feature learning approaches may be required to better exploit autonomic signals in multi-class sleep disorder classification.

From a clinical perspective, these results suggest that MAF-SleepNet can serve as a valuable adjunct to expert scoring, reducing the manual burden of PSG analysis and accelerating the diagnostic process. Nevertheless, caution is warranted in cases of respiratory and motor disorders, where additional clinical validation and multimodal integration beyond EEG, EOG, and EMG may be required to improve classification reliability further. Overall, the framework’s strong performance for cortical and hypersomnolence-related disorders, coupled with its robustness in identifying healthy cases, supports its potential to enhance clinical workflows and facilitate more efficient diagnosis of sleep disorders.

### 5.3. Limitations and Future Directions

The present study offers several notable strengths that advance the state of the art in automatic sleep disorder classification. By explicitly adopting the ICSD-3 taxonomy [10], the framework ensured clinically meaningful class definitions and included only PSG-requiring disorders. The integration of three distinct datasets enhanced heterogeneity and broadened demographic coverage, while the rigorous enforcement of subject-independent evaluation protocols provided a reliable assessment of generalization performance. Furthermore, the proposed MAF-SleepNet architecture, which leverages multimodal attention-enhanced fusion, demonstrated substantial performance improvements over unimodal configurations and achieved near-perfect separation of healthy subjects. These features collectively highlight the model’s potential as a clinically relevant tool for supporting expert diagnosis. Nevertheless, despite these advantages, several limitations remain that should be addressed in future work, as outlined below.

- In this study, we evaluated the proposed MAF-SleepNet on a dataset created by combining three publicly available PSG databases, which improved heterogeneity and broadened demographic coverage. Data augmentation strategies were also employed to mitigate the risk of overfitting and enhance model robustness. Nevertheless, the overall sample size remains limited, and PSG recordings are inherently high-dimensional, often spanning hundreds of megabytes per subject. Such large-scale data increases computational demands and exacerbates the risk of overfitting when the number of available subjects is restricted. In particular, disorders such as PLMD and OSA were represented by relatively few recordings, which likely contributed to lower classification performance for these categories and constrained generalizability. Expanding the dataset to include larger, more balanced cohorts across diverse clinical populations, ideally through multicenter collaborations, will be essential to further strengthen the reliability and clinical applicability of the proposed framework. In addition, all signals were resampled to 128 Hz to ensure a unified temporal resolution across datasets. Although this may reduce high-frequency components, particularly in ECG signals, resampling was performed using MATLAB’s resample function with built-in anti-aliasing filtering, minimizing spectral distortion. This reflects a trade-off between cross-dataset consistency and signal fidelity. Moreover, the collection and public release of additional large-scale PSG datasets would be highly beneficial for advancing generalizability and enabling more systematic benchmarking across studies.
- A further limitation arises from the inclusion of both pediatric (NCH-SDB) and adult (CAPSD and ANPHY) datasets, which introduces age-related heterogeneity. Since augmented samples are derived from the same subjects, age-related patterns are preserved and cannot be treated as an independent factor. Consequently, age-stratified analysis was not feasible given the limited number of subjects in the evaluation setting (e.g., 42 subjects in the test set). Although subject-independent evaluation reduces subject-specific bias, potential age-related confounding cannot be entirely excluded. Future work will focus on age-aware modeling and stratified evaluation on larger cohorts.
- While multimodal fusion improved overall performance, ECG did not provide the expected benefit for OSA and, in some cases, even reduced accuracy. A permutation-based feature importance analysis on the ECG-only configuration of the base model indicated that, although ECG features contributed to classification performance, their impact remained limited and was not uniformly observed across different classes. This suggests that the extracted ECG features may not fully capture the complex and heterogeneous autonomic patterns associated with different sleep disorders. Furthermore, the absence of direct respiratory channels, such as airflow, thoracic/abdominal effort, and oxygen saturation, limited the classification of respiratory and motor disorders. Future work should integrate respiratory signals and more advanced ECG representations to address these limitations.
- Although combining three databases improved heterogeneity, the study remains limited to retrospective data and has not yet been validated on an independent external dataset or in prospective or real-world clinical settings. A leave-one-dataset-out test was not feasible, as each source contributes different diagnostic classes; nonetheless, the combined multi-site, multi-device cohort already provides cross-site heterogeneity beyond the single-dataset protocols used in prior work. External validation across multiple centers, recording systems, and diverse patient populations will be essential to establish the true clinical robustness and generalizability of the proposed framework.
- Although the model achieved high performance, it remains largely a “black box,” which may hinder clinician trust and adoption. Incorporating explainable AI techniques [82] that provide transparent and physiologically meaningful insights will be important for facilitating clinical acceptance.
- The framework was restricted to five PSG-requiring categories defined by the ICSD-3, which, although clinically meaningful, do not capture the full spectrum of sleep disorders. Expanding the taxonomy to include additional disorders within the same categories would increase diagnostic coverage and clinical utility. Moreover, the current model assumes a single primary diagnosis per patient and cannot yet account for comorbid sleep disorders, which are frequently encountered in clinical practice. Future work should therefore focus on extending the framework to multi-label classification, enabling the simultaneous identification of multiple co-occurring disorders.
- The current framework relies on handcrafted or pre-processed features extracted from PSG signals before being fed into the network. While this strategy reduces dimensionality and facilitates model training, it may also limit the ability of the system to capture subtle temporal- or frequency-domain patterns present in the raw signals, including transient events substantially shorter than the feature-extraction window. Future work could explore end-to-end learning directly from raw waveforms using advanced techniques such as transformers [83,84] or hybrid models [85,86].

Collectively, addressing these limitations through larger and more diverse datasets, integration of additional physiological channels, enhanced interpretability, and expanded diagnostic scope will be critical to translating the proposed framework into a clinically reliable tool for routine practice.

## 6. Conclusions

This study introduced MAF-SleepNet, a novel multimodal attention-enhanced fusion network for the automatic classification of multiple sleep disorders using PSG recordings. Unlike most prior works that focused on a single disorder, relied on a single dataset, or employed labeling strategies that introduced considerable label noise, the proposed framework explicitly adhered to the ICSD-3 taxonomy, included only PSG-requiring disorders, and analyzed entire overnight recordings as temporally ordered sequences. By integrating EEG, EOG, and leg EMG signals through modality-specific attention and cross-modal fusion, MAF-SleepNet effectively captured both intra- and inter-modality dependencies, enabling richer and clinically meaningful representations of sleep pathophysiology. Experimental results demonstrated strong performance and generalizability, with 88.41% accuracy and 86.89% macro-F1 under subject-independent hold-out evaluation, 86.07 ± 3.66% accuracy and 82.67 ± 4.46% macro-F1 under subject-independent cross-validation, and near-ceiling scores under subject-dependent evaluation. The model consistently achieved perfect separation of healthy subjects and showed robust performance for cortical and hypersomnolence-related disorders such as NFLE and CDHs, while performance was moderate for RBD and comparatively weaker for episodic motor and respiratory disorders including PLMD and OSA, reflecting their greater clinical complexity. These findings highlight both the potential and the challenges of automated multimodal analysis in sleep disorder diagnosis. In particular, two methodological advances stand out: first, the use of whole-night analysis, which reduces label noise caused by the episodic nature of sleep disorders, and second, the adoption of adaptive information fusion, which has proven more effective than unimodal or loosely coupled multimodal approaches. Future work should prioritize the creation of larger and more diverse publicly available PSG datasets, the integration of respiratory and advanced ECG-derived features, prospective validation in clinical settings, and the development of explainable and multi-label models to address comorbidities better and support clinical adoption.

## Figures and Tables

**Table 1:** Summary of existing studies on multi-class sleep disorder classification using conventional ML and DL.

Ref	Classes	Dataset	# Sub	Input Signal	Method	Results (%)			
						Acc	Sen	Spe	F1
[19], 2023	Bruxism, insomnia, narcolepsy, NFLE, SBD, PLMD, RBD, healthy	CAPSD	108	EEG, ECG, EMG	10-s windowing, and MML-DMS: six independent CNNs + feature concatenation + MLP	99.1	n/a	n/a	99.0
[42], 2022	Bruxism, insomnia, narcolepsy, NFLE, SBD, PLMD, RBD, healthy	CAPSD	108	EEG, EMG, EKG, EOG	5-s windowing, spectrogram conversion using STFT, and DL-R: four 5-layer CNNs.	n/a	>95	>95	n/a
[20], 2022	Insomnia, narcolepsy, NFLE, PLMD, RBD, healthy	CAPSD	66	EMG EOG	30-s windowing, sub-band decomposition using BMMFB, Hjorth parameters FE, and EBT.	94.3	-	-	-
[43], 2021	Insomnia, NFLE, PLMD, RBD, healthy	CAPSD	35	ECG	10-s windowing, SDN: 1D-CNN + GRU.	-	-	-	>95
[39], 2021	Insomnia, narcolepsy, NFLE, SBD, PLMD, RBD, healthy	CAPSD	77	EEG	30-s windowing, sub-band decomposition using THFB, extraction of Hjorth parameters, and EBoT.	91.3	n/a	n/a	n/a
[40], 2021	Bruxism, insomnia, narcolepsy, NFLE, SBD, PLMD, RBD, healthy	CAPSD	107	EEG	30-s windowing, two types of CFC FE, and RF.	74.0	59.1	n/a	64.9
[38], 2020	Insomnia, SBD, RBD, healthy	CAPSD	51	ECG	30-s windowing, spectral FE, DT-based SVM for sleep stage classification, sleep quality FE, and EBT.	86.3	84.1	94.2	n/a
[41], 2018	OSA, hypopnea, CSA, sleep stages	MIT-BIH	16	EEG	30-s windowing, FE using PSD, PCA, PLS, and SVM.	85.0	n/a	n/a	n/a

#: number of; Acc: accuracy; BMMFB: bi-orthogonal maximally flat multiplier-less wavelet filter bank; CAPSD: CAP sleep database; CFC: cross-frequency coupling; CNN: convolutional neural network; CSA: central sleep apnea; DT: decision tree; EBoT: ensemble boosted tree; EBT: ensemble bagged tree; ECG: electrocardiography; EEG: electroencephalography; F1: F1-score; FE: feature extraction; n/a: not available; GRU: gated recurrent unit; MML-DMS: multimodal and multilabel decision-making system; multi-layer perceptron; NFLE: nocturnal frontal lobe epilepsy; OSA: obstructive sleep apnea; PCA: principal component analysis; PLMD: periodic limb movement disorder; PLS: partial least squares; PSD: power spectral density; RBD: REM sleep behavior disorder; RF: random forest; Sen: sensitivity; SBD: sleep-related breathing disorder; SDN: sleep disorder network; Spe: specificity; STFT: short-time Fourier transform; SVM: support vector machine; THFB: triplet half-band filter bank.

**Table 2:** Summary of the datasets used in this study, the number of recordings selected from each, and their corresponding diagnoses.

Dataset	Recordings Used	Diagnoses
CAPSD [48,49]	59	22 RBD, 37 NFLE
NCH-SDB [48,51,52]	59	15 CDH, 24 OSA, 20 PLMD
ANPHY [53]	23	23 Healthy
Combined dataset	141	-

**Table 3:** Bandpass filter cut-off frequencies applied to each physiological signal according to AASM guidelines [17,18].

Signal	Lower Cut-Off (Hz)	Upper Cut-Off (Hz)
EEG	0.3	35
EOG	0.3	35
EMG	10	100
ECG	0.3	100

**Table 4:** Extracted epoch-level features for each physiological modality.

Modality	Features	Count
EEG	δ, θ, α, β relative powers [56], spectral entropy [57], Shannon entropy [55], line length [58], RMS [58], zero-crossings [57], Hjorth mobility & complexity [20]	11
EOG	RMS [58], peak-to-peak amplitude; zero-crossings [57], line length [58], Shannon entropy [55]	5
Chin EMG	RMS [58], peak-to-peak amplitude [59], zero-crossings [57], movement index (adaptive threshold), Shannon entropy [55]	5
Leg EMG	RMS [58], peak-to-peak amplitude [59]; zero-crossings [57], movement index (adaptive threshold), Shannon entropy [55]	5
ECG	Variance [60], peak-to-peak amplitude [59], RR interval variability [61], Shannon entropy [55], line length [58]	5

**Table 5:** Overview of the original (the combined dataset) and the augmented datasets.

Diagnosis	Recordings	Augmentation Factor	Augmented Sequences
RBD	22	30	660
NFLE	37	30	1110
CDH	15	30	450
OSA	24	30	720
PLMD	20	30	600
Healthy	23	30	690
Total	141	-	4230

**Table 6:** Subject-independent hold-out partitioning of the augmented dataset into training and test subsets, with both the number of original recordings and corresponding augmented sequences (recordings/sequences).

Diagnosis	Train	Test	Total
RBD	17/510	5/150	22/660
NFLE	20/600	17/510	37/1110
CDH	11/330	4/120	15/450
OSA	18/540	6/180	24/720
PLMD	15/450	5/150	20/600
Healthy	18/540	5/150	23/690
Total	99/2970	42/1260	141/4230

**Table 7:** Summary of hyperparameters employed in all experiments.

Hyperparameter	Value
Optimizer	Adam
Learning rate	0.0001
Batch size	32
Kernel size	5
# Filters in RFEB	96
Loss function	Cross-entropy
Max epochs	20
Shuffle	Per epoch
Sequence padding direction	Left
Validation patience	5 epochs

#: number of.

**Table 8:** Final architecture of the proposed MAF-SleepNet.

Input Modalities	# RFEBs in MSFEB	# RFEBs After Fusion	Attention Level
EEG, EOG, Leg EMG	EEG: 3 (1 RFEB-A + 2 RFEB-B) EOG: 3 (1 RFEB-A + 2 RFEB-B) Leg EMG: 2 (1 RFEB-A + 1 RFEB-B)	None	Branch-level attention (after each MSFEB)
Total trainable parameters (MAF-SleepNet)		293.4 k	

#: number of.

**Table 9:** Classification performance of the proposed MAF-SleepNet under the subject-independent hold-out evaluation.

Class	Performance Metrics (%)				
	Sen.	Spe.	Pre.	F1	Acc.
NFLE	93.53	94.13	91.55	92.53	-
OSA	83.33	99.63	97.40	89.82	-
RBD	85.33	95.59	72.32	78.29	-
PLMD	78.67	95.59	70.66	74.45	-
CDH	75.83	100	100	86.26	-
Healthy	100	100	100	100	-
Macro-average	86.12	97.49	88.66	86.89	Overall: 88.41

**Table 10:** Classification performance of the proposed MAF-SleepNet under subject-independent 4-fold cross-validation.

Class	Performance Metrics (%)			
	Sen.	Spe.	Pre.	F1
NFLE	98.59 ± 1.64	96.98 ± 2.00	94.16 ± 3.59	96.27 ± 1.21
OSA	63.34 ± 2.61	98.43 ± 1.29	88.25 ± 9.00	73.64 ± 4.32
RBD	89.31 ± 15.54	96.45 ± 0.69	81.17 ± 5.80	84.83 ± 10.31
PLMD	68.00 ± 6.62	94.69 ± 1.93	65.20 ± 10.24	66.50 ± 8.38
CDH	79.58 ± 16.40	96.81 ± 0.83	73.17 ± 6.70	75.88 ± 10.89
Healthy	97.92 ± 4.17	100.0 ± 0.00	100.0 ± 0.00	98.91 ± 2.18
Macro-average	82.79 ± 5.18	97.23 ± 0.75	83.66 ± 4.06	82.67 ± 4.46
95% CI	74.55–91.03	96.04–98.42	77.20–90.12	75.57–89.77
Overall Accuracy		86.07 ± 3.66 (95% CI: 80.25–91.89)		

**Table 11:** Classification performance of the proposed MAF-SleepNet under subject-dependent 4-fold cross-validation.

Class	Performance Metrics (%)			
	Sen.	Spe.	Pre.	F1
NFLE	100.0 ± 0.00	99.49 ± 0.63	98.60 ± 0.63	99.29 ± 0.87
OSA	96.67 ± 2.36	100.0 ± 0.00	100.0 ± 0.00	98.30 ± 1.22
RBD	97.88 ± 2.50	100.0 ± 0.00	100.0 ± 0.00	98.92 ± 1.28
PLMD	100.0 ± 0.00	99.23 ± 0.30	95.57 ± 0.30	97.73 ± 0.87
CDH	98.67 ± 2.66	100.0 ± 0.00	100.0 ± 0.00	99.32 ± 1.37
Healthy	100.0 ± 0.00	100.0 ± 0.00	100.0 ± 0.00	100.0 ± 0.00
Macro-average	98.87 ± 0.65	99.79 ± 0.14	99.03 ± 0.51	98.93 ± 0.60
95% CI	97.84–99.90	99.57–100.0	98.22–99.84	97.98–99.88
Overall Accuracy		98.96 ± 0.66 (95% CI: 97.91–100.0)		

**Table 12:** Performance of the base model with different input modality configurations. Results of the three best-performing configurations (T1, T6, Q4) are highlighted in bold.

ID	Input Modalities					Performance Metrics (%)		# Trainable Params.
	EEG	ECG	EOG	Leg EMG	Chin EMG	Acc.	Macro F1	
S1	✔	-	-	-	-	71.19	67.17	104.45 k
S2	-	✔	-	-	-	47.18	36.74	98.69 k
S3	-	-	✔	-	-	68.69	59.57	
S4	-	-	-	✔	-	58.04	49.04	
S5	-	-	-	-	✔	64.05	49.88	
D1	✔	✔	-	-	-	54.09	49.40	203.14 k
D2	✔	-	✔	-	-	74.61	71.55	
D3	✔	-	-	✔	-	79.06	75.00	
D4	✔	-	-	-	✔	70.93	63.92	
**T1**	✔	-	✔	✔	-	**81.25**	**78.56**	301.83 k
T2	✔	✔	✔	-	-	65.08	61.18	
T3	✔	✔	-	✔	-	66.01	56.40	
T4	✔	-	✔	-	✔	75.02	69.87	
T5	✔	✔	-	-	✔	67.05	59.17	
**T6**	✔	-	-	✔	✔	**81.35**	**78.73**	
Q1	✔	✔	✔	✔	-	71.48	64.96	400.52 k
Q2	✔	✔	✔	-	✔	71.07	62.62	
Q3	✔	✔	-	✔	✔	74.01	68.65	
**Q4**	✔	-	✔	✔	✔	**80.24**	**77.10**	
All	✔	✔	✔	✔	✔	74.79	71.02	499.21 k

#: number of, ✓: modality included in the configuration.

**Table 13:** Permutation-based feature importance analysis of ECG features in the ECG-only configuration (S2).

Feature	Macro-F1 Drop	Std
Variance	0.1035	0.0080
Peak-to-Peak Amplitude	0.1374	0.0079
RR Interval Variability	0.1102	0.0087
Shannon Entropy	0.0943	0.0063
Line Length	0.1348	0.0071

**Table 14:** Performance of attention strategies across the three best configurations. The best results for each configuration are highlighted in bold.

Config.	No Attention	Branch-Level Attention	Fusion-Level Attention	Combined Attention
**T1**	Acc:83.02 F1:79.18 **#** Params:255.4 k	Acc:**88.41** F1:**86.89** **# Params:293.4 k**	Acc:74.76 F1:64.98 # Params:293.3 k	Acc:85.40 F1:77.85 # Params:331.3 k
**T6**	Acc:81.99 F1:77.27 # Params:301.8 k	Acc:83.57 F1:80.12 # Params:339.8 k	Acc:**85.79** F1:**83.41** **# Params:339.6 k**	Acc:71.91 F1:64.02 # Params:377.6 k
**Q4**	Acc:**84.71** F1:**83.63** **# Params:493.2 k**	Acc:76.59 F1:69.81 # Params:543.9 k	Acc:77.94 F1:72.45 # Params:543.6 k	Acc:73.23 F1:65.08 # Params:594.3 k

#: number of.

**Table 15:** Comparison of proposed MAF-SleepNet with existing methods on CAPSD and under different evaluation settings.

Ref	Dataset	Method	Evaluation	Performance Metrics (%)			
				Acc	Sen	Spe	F1
[19], 2023	CAPSD	MML-DMS: six independent CNNs + feature concatenation + MLP	Subject-dependent	99.1	n/a	n/a	99.0
[42], 2022	CAPSD	DL-R: four 5-layer CNNs.	Subject-dependent	n/a	>95	>95	n/a
[20], 2022	CAPSD	Handcrafted FE and EBT.	Subject-dependent	94.3	-	-	-
[43], 2021	CAPSD	SDN: 1D-CNN + GRU.	Subject-dependent	-	-	-	>95
[39], 2021	CAPSD	Handcrafted FE and EBoT.	Subject-dependent	91.3	n/a	n/a	n/a
[40], 2021	CAPSD	FE and RF.	Subject-dependent	74.0	59.1	n/a	64.9
[38], 2020	CAPSD	Handcrafted FE and EBT.	Subject-dependent	86.3	84.1	94.2	n/a
Ours	Combined three datasets	MAF-SleepNet	4-fold Subject-dependent	98.96	98.87	99.79	98.93
Ours	CAPSD	MAF-SleepNet	4-fold Subject-dependent	99.38	99.10	99.85	99.50
Ours	Combined three datasets	MAF-SleepNet	4-fold Subject-independent	86.07	82.79	97.23	82.67

n/a: not available.

**Table 16:** Comparison of MAF-SleepNet with modern DL baselines.

**Model**	Accuracy (%)	Macro-F1 (%)	# Trainable Params	McNemar’s Test
1D-CNN	78.02	73.56	290.8 k	$\chi^{2}\left(1\right)=56.52,\;p<0.001$
CNN-BiLSTM	83.02	76.75	179.7 k	$\chi^{2}\left(1\right)=25.51,\;p<0.001$
Transformer	70.44	67.84	151.4 k	$\chi^{2}\left(1\right)=164.18,\;p<0.001$
MAF-SleepNet	88.41	86.89	293.4 k	

#: number of.

**Table 17:** Class-specific permutation-based feature importance analysis for OSA (ECG-only configuration).

Feature	Recall Drop
Variance	−0.0005
Peak-to-Peak Amplitude	0.1569
RR Interval Variability	0.0906
Shannon Entropy	0.3119
Line Length	−0.0100

## Data Availability

The original contributions presented in this study are included in the article/Supplementary Materials. Further inquiries can be directed to the corresponding author.

## Supplementary Materials

The following supporting information can be downloaded at: https://www.mdpi.com/article/10.3390/diagnostics16152317/s1, Section S1: Detailed description of the datasets used in this study; Section S1.1: CAP Sleep Database (CAPSD); Table S1: Details of the selected recordings from CAPSD [48,49]; Section S1.2: Nationwide Children’s Hospital Sleep DataBank (NCH-SDB); Table S2: Details of the selected OSA recordings from NCH-SDB [48,51,52] for the SBD category; Table S3: Details of the selected PLMD recordings from NCH-SDB [48,51,52] for the SMD category; Table S4: Details of the selected CDH recordings from NCH-SDB [48,51,52]; Section S1.3: ANPHY-Sleep Dataset (ANPHY); Table S5: Details of the selected recordings from ANPHY [53]; Section S2: Mathematical definitions of the extracted features; Table S6: Detailed mathematical definitions of all extracted features; Section S3: Architectural details of the deep learning baselines. Ref. [87] is cited in Supplementary Materials.

## Abbreviations

The following abbreviations are used in this manuscript:

AASM	American Academy of Sleep Medicine
Acc.	Accuracy
AI	Artificial Intelligence
ANPHY	ANPHY-Sleep Dataset
CAP	Cyclic Alternating Pattern
CAPSD	CAP Sleep Database
CC BY	Creative Commons Attribution
CDH	Central Disorders of Hypersomnolence
CDHs	Central Disorders of Hypersomnolence
CNN	Convolutional Neural Network
CNNs	Convolutional Neural Networks
Conv1D	One-Dimensional Convolution
CRSWDs	Circadian Rhythm Sleep–Wake Disorders
CSA	Central Sleep Apnea
DL	Deep Learning
DT	Decision Tree
EBT	Ensemble Bagged Tree
EBoT	Ensemble Boosted Tree
ECG	Electrocardiography
EEG	Electroencephalography
EMG	Electromyography
EOG	Electrooculography
F1	F1-Score
FCL	Fully Connected Layer
FE	Feature Extraction
GAP	Global Average Pooling
GRU	Gated Recurrent Unit
Hz	Hertz
ICSD-3	International Classification of Sleep Disorders, Third Edition
MAF-SleepNet	Multimodal Attention-Enhanced Fusion Network
MHSA	Multi-Head Self-Attention
MIT-BIH	Massachusetts Institute of Technology–Beth Israel Hospital
ML	Machine Learning
MLP	Multi-Layer Perceptron
MML-DMS	Multimodal and Multilabel Decision-Making System
MSFEB	Modality-Specific Feature Extractor Branch
NCH-SDB	Nationwide Children’s Hospital Sleep DataBank
NFLE	Nocturnal Frontal Lobe Epilepsy
OSA	Obstructive Sleep Apnea
PCA	Principal Component Analysis
PLM	Periodic Limb Movement
PLMD	Periodic Limb Movement Disorder
PLS	Partial Least Squares
Pre.	Precision
PSG	Polysomnography
PSD	Power Spectral Density
RBD	REM Sleep Behavior Disorder
REM	Rapid Eye Movement
RF	Random Forest
RFEB	Residual Feature Extractor Block
RFEB-A	Residual Feature Extractor Block-A
RFEB-B	Residual Feature Extractor Block-B
RLS	Restless Legs Syndrome
RMS	Root Mean Square
RNN	Recurrent Neural Network
RR	R-R Interval
SBD	Sleep-Related Breathing Disorder
SBDs	Sleep-Related Breathing Disorders
SDN	Sleep Disorder Network
Sen.	Sensitivity
SGDM	Stochastic Gradient Descent with Momentum
SMDs	Sleep-Related Movement Disorders
Spe.	Specificity
STFT	Short-Time Fourier Transform
SVM	Support Vector Machine
SVMs	Support Vector Machines
S-W	Sleep–Wake
