# Supplementary material for: MAF-SleepNet: A Multimodal Attention-Enhanced Fusion Network for Automatic Multi-Class Sleep Disorder Classification from Polysomnography
Source: Diagnostics (Basel). 2026 Jul 23;16(15):2317. doi: 10.3390/diagnostics16152317 (PMC13465423; doi:10.3390/diagnostics16152317)
Supplement: Supplementary file 1 [file diagnostics-16-02317-s001.zip › diagnostics-4366020-supplementary.pdf]

Section S1. Detailed Dataset Descriptions and Selected Recordings

This section provides detailed information on the individual recordings selected from the three datasets used in this study.

Section S1.1. CAP Sleep Database (CAPSD)

The CAPSD [1,2] is a publicly available collection of 108 full-night PSG recordings, each lasting approximately 9–10 hours, acquired at the Sleep Disorders Center of the Ospedale Maggiore of Parma, Italy. Of these, 16 recordings were obtained from healthy sleepers without any neurological disorders, while the remaining 92 were from patients diagnosed with various sleep disorders: 40 with NFLE, 22 with RBD, 10 with PLMD, 9 with insomnia, 5 with narcolepsy, 4 with SBDs, and 2 with bruxism. Each recording includes at least three EEG channels (F3 or F4, C3 or C4, and O1 or O2, referenced to A1 or A2), two EOG channels, submental EMG, bilateral anterior tibial EMG, ECG, and respiration signals with sampling rates ranging from 100 Hz to 512 Hz, as well as additional bipolar EEG derivations. As described earlier, insomnia was excluded from the overall classification scheme; therefore, recordings of patients with insomnia were also excluded from CAPSD. Furthermore, other disorders with very few available recordings in this dataset, such as bruxism, SBDs, and narcolepsy, were also excluded to maintain consistency with the different datasets used in this study and to avoid extreme class imbalance. Consequently, 22 recordings from patients with RBD and 37 recordings from patients with NFLE were selected from CAPSD. The details of these selected recordings are provided in **Table S1.**

Table S1. Details of the selected recordings from CAPSD [1,2].

**Diagnosis**	**Total records**	**Included**	**Age (mean ±SD)**	**Sex (M/F)**	**Duration (h, mean ±SD)**	**Excluded**	**Excluded IDs**
RBD	22	22	70.73 ± 6.39	19/3	9.72 ± 2.11	0	-
NFLE	40	37	30.46 ± 10.88	19/18	8.85 ± 0.87	3	nfle8, nfle25, nfle33

Section S1.2. Nationwide Children's Hospital Sleep DataBank (NCH-SDB)

The NCH-SDB [1,3,4] is a large-scale, publicly available dataset hosted on PhysioNet, containing 3,984 pediatric PSG recordings collected from 3,673 patients at the Nationwide Children's Hospital Sleep Center in Columbus, Ohio, USA, between 2017 and 2019. Each PSG includes multiple EEG channels, bilateral EOG, chin and tibial EMG, ECG, nasal and oral airflow, thoracic and abdominal respiratory effort, SpO_2, end-tidal CO_2, snoring sounds, and body position, with sampling rates ranging from 256 Hz to 512 Hz. The dataset is accompanied by rich clinical and demographic metadata, including age, sex, height, weight, body mass index, and relevant medical history. In this study, only recordings from patients diagnosed with SBDs (specifically OSA), SMDs (PLMD), and CDHs (narcolepsy type 1, narcolepsy type 2, or hypersomnia unspecified) were included. To ensure consistency with the other datasets used, recordings corresponding to underrepresented disorders or non-target ICSD-3 categories were excluded. Additionally, using the Diagnosis.csv file, only entries with DX_SOURCE_TYPE marked as Final Dx were retained, and patients assigned to multiple major sleep disorder

categories were excluded. Consequently, 15 recordings with CDH, 24 with OSA, and 20 with PLMD were selected from NCH-SDB. The details of these selected recordings are provided in **Tables S2, S3, and S4**.

Table S2. Details of the selected OSA recordings from NCH-SDB [1,3,4] for the SBD category.

**Subjects ID**	**Age**	**Sex**	**Duration (h)**
2137_12121	2.75	M	8.67
14626_7642	13.83	M	10.31
1408_16276	16.85	M	10.75
11323_21685	3.83	M	11.45
9073_11623	3.02	F	11.32
6814_21103	10.86	M	8.38
16981_5875	15.06	M	8.62
17020_16600	5.26	F	10.22
15970_14674	8.25	F	9.03
4135_15835	4.81	F	10.16
9709_5653	3.10	M	9.25
9709_25444	3.48	M	11.30
433_10945	2.54	M	11.41
17752_19516	7.03	F	10.16
11257_20695	13.25	F	10.12
12145_24604	4.75	F	10.43
8317_4678	8.79	F	10.11
16582_3874	14.93	F	11.83
12571_18946	6.57	M	9.45
11506_781	11.10	F	8.96
20320_12025	10.67	M	10.34
1693_685	14.23	F	10.74
12295_17314	21.48	M	11.02
18082_9487	10.52	M	10.93
**Summary** (n=24)	9.04 ± 5.16	13 M/11 F	10.21 ± 0.97

Table S3. Details of the selected PLMD recordings from NCH-SDB [1,3,4] for the SMD category.

Subjects ID	Age	Sex	Duration (h)
18676_12235	13.82	F	9.74
1372_26035	9.50	M	9.35
7657_14263	17.56	M	9.42
13072_26239	8.76	F	10.20
2725_26161	5.52	F	7.88
17833_8983	3.13	M	11.57
11269_8845	9.18	M	10.20
5953_25189	12.10	M	9.66
17659_23386	9.66	M	11.42
11908_7534	5.39	F	11.01
15088_10261	18.86	M	9.03
16003_18829	14.00	F	12.21
18793_17224	10.25	F	11.11
17566_12625	9.65	M	9.39
14182_22006	10.73	F	9.61
16912_18493	8.35	M	9.59
16126_15514	11.15	F	10.09
19780_11713	15.84	M	11.10
12895_9994	6.12	F	11.15
16876_5509	15.13	F	9.07
**Summary** (n=20)	10.74 ± 4.07	10 M/10 F	10.14 ± 1.04

Table S4. Details of the selected CDH recordings from NCH-SDB [1,3,4].

Subjects ID	Age	Sex	Duration (h)
5320_22120	10.88	F	9.13
8443_12766	12.67	M	9.63
17647_12985	11.52	F	10.18
4747_439	16.94	M	11.72
18523_7843	1345	F	10.78
10165_11332	16.76	F	10.49
8398_26014	17.05	M	12.20
5440_24349	18.02	F	9.35
19504_18289	12.80	M	9.71
14395_262	15.63	M	11.84
15433_8161	17.65	F	9.71
6298_9445	12.00	M	10.06
163_20485	13.80	F	9.17
7006_4264	13.10	F	11.33
13342_20593	13.13	M	8.81
**Summary** (n=15)	14.36 ± 2.	7 M/8 F	10.27 ± 1.04

Section S1.3. ANPHY-Sleep Dataset (ANPHY)

The ANPHY [5] is a publicly available dataset hosted on the Open Science Framework, comprising overnight PSG recordings from 29 healthy adults collected at the Montreal Neurological Institute, Canada. Each recording includes high-density EEG (83 electrodes placed according to the 10-10 system), bilateral EOG, chin EMG, bilateral anterior tibialis EMG, and ECG channels, with a sampling rate of 1000 Hz. Since this dataset exclusively contains healthy participants, it was used to represent the healthy class in this study. To maintain a balanced class distribution across datasets and avoid substantial class imbalance in the combined dataset, 23 recordings were selected. The details of these selected recordings are provided in **Table S5**.

Table S5. Details of the selected recordings from ANPHY [5].

Diagnosis	Total records	Included	Age (mean ±SD)	Sex (M/F)	Duration (h, mean ±SD)	Excluded	Excluded IDs
Healthy	29	23	31.39 ± 5.97	15/8	6.95 ± 0.81	6	EBCTL21, EBCTL25, EBCTL26, EBCTL27, EBCTL28, EBCTL29

Section S2. Detailed mathematical definitions of extracted features

This section provides detailed information on the extracted features. All features were extracted from consecutive 15-second epochs of each overnight PSG recording. Signals were resampled to a common sampling frequency of f_s = 128 Hz. For EEG, power spectral density (PSD) estimates were obtained using Welch's method.

Table S6. Detailed mathematical definitions of all extracted features.

Feature	Signal	Equation	Description	Ref.
Relative Power δ	EEG	$\boldsymbol{RP_{\delta}} = \dfrac{\boldsymbol{P_{0.5-4}}}{\boldsymbol{P_{total}}}$	$\boldsymbol{P_{0.5-4}}$: *power in 0.5-4 Hz;* $\boldsymbol{P_{total}}$: *total spectral power*	[6]
Relative Power θ	EEG	$\boldsymbol{RP_{\theta}} = \dfrac{\boldsymbol{P_{4-8}}}{\boldsymbol{P_{total}}}$	$\boldsymbol{P_{4-8}}$: *power in 4-8 Hz*	[6]
Relative Power α	EEG	$\boldsymbol{RP_{\alpha}} = \dfrac{\boldsymbol{P_{8-13}}}{\boldsymbol{P_{total}}}$	$\boldsymbol{P_{8-13}}$: *power in 8-13 Hz;*	[6]
Relative Power β	EEG	$\boldsymbol{RP_{\beta}} = \dfrac{\boldsymbol{P_{13-30}}}{\boldsymbol{P_{total}}}$	$\boldsymbol{P_{13-30}}$: *power in 13-30 Hz;*	[6]
Spectral Entropy	EEG	$\boldsymbol{H_{\mathrm{spec}}} = -\sum_{i} \boldsymbol{p_i} \log_2 \boldsymbol{p_i}$	$\boldsymbol{p_i}$ *is the normalized power of the i-th frequency bin*	[7]
Shannon Entropy	EEG, EOG, EMG ECG	$\boldsymbol{H_{\mathrm{wav}}} = -\sum_{k} \boldsymbol{q_k} \log_2 \boldsymbol{q_k}$	$\boldsymbol{q_k}$*is the normalized energy of the k-th wavelet coefficient*	[8]
Line Length	EEG, EOG, ECG	$\boldsymbol{LL} = \log\left(\sum_{m=1}^{M-1} \lvert \boldsymbol{x}[\boldsymbol{m}] - \boldsymbol{x}[\boldsymbol{m}-1] \rvert\right)$	$\boldsymbol{x}[\boldsymbol{m}]$: *signal amplitude at sample* $\boldsymbol{m}$; $\boldsymbol{M}$: *number of samples in the epoch*	[9]
RMS	EEG, EOG, EMG	$\boldsymbol{RMS} = \sqrt{\dfrac{1}{\boldsymbol{M}} \sum_{m=1}^{M} \boldsymbol{x}[\boldsymbol{m}]^2}$	$\boldsymbol{x}[\boldsymbol{m}]$: *signal amplitude at sample* $\boldsymbol{m}$	[9]
Zero-crossings	EEG, EOG, EMG	$\boldsymbol{ZC} = \sum_{m=1}^{M-1} \mathbf{1}[\boldsymbol{x}[\boldsymbol{m}].\boldsymbol{x}[\boldsymbol{m}+1] < 0]$	$\boldsymbol{x}[\boldsymbol{m}]$: *signal amplitude at sample* $\boldsymbol{m}$	[7]

Table S6. (continued).

Hjorth Mobility	EEG	$\boldsymbol{m} = \frac{\boldsymbol{\sigma_{\Delta x}}}{\boldsymbol{\sigma_x}}$	$\boldsymbol{\sigma_x}$: standard deviation of the signal; $\boldsymbol{\sigma_{\Delta x}}$: standard deviation of the first derivative of the signal within the epoch	[10]
Hjorth Complexity	EEG	$\boldsymbol{c} = \frac{\boldsymbol{\sigma_{\Delta^2 x}} / \boldsymbol{\sigma_{\Delta x}}}{\boldsymbol{m}}$	$\boldsymbol{\sigma_{\Delta^2 x}}$: standard deviation of the second derivative	[10]
Peak-to-Peak Amplitude	EOG, EMG, ECG	$\boldsymbol{PTP} = \boldsymbol{max}(\boldsymbol{x}[\boldsymbol{m}]) - \boldsymbol{min}(\boldsymbol{x}[\boldsymbol{m}])$		[11]
Movement Index	EMG	$\boldsymbol{MI} = \sum_{\boldsymbol{m}=1}^{\boldsymbol{M}} \mathbf{1}[\boldsymbol{x}[\boldsymbol{m}] > \boldsymbol{T}]$	$\boldsymbol{T}$: threshold, defined as 7.5 times the median amplitude of the epoch.	-
Variance	ECG	$\boldsymbol{Var} = \frac{\mathbf{1}}{\boldsymbol{M}} \sum_{\boldsymbol{m}=1}^{\boldsymbol{M}} (\boldsymbol{x}[\boldsymbol{m}] - \boldsymbol{\mu_x})^2$	$\boldsymbol{\mu_x}$: mean of the signal within the epoch	[12]
RR Interval Variability	ECG	$\boldsymbol{RR_{var}} = \boldsymbol{std}\left(\left\{\frac{\boldsymbol{locs_{k+1}} - \boldsymbol{locs_k}}{\boldsymbol{f_s}} \middle\| \boldsymbol{k} = \mathbf{1}, \mathbf{2}, \dots, \boldsymbol{K} - \mathbf{1}\right\}\right)$	$\boldsymbol{locs_k}$: index of the $\boldsymbol{k-th}$ R peak in the ECG epoch; $\boldsymbol{K}$: number of detected R peaks in the epoch	[13]

Section S3. Architectural details of the deep learning baselines

This section provides detailed information on the three deep learning baselines used to benchmark MAF-SleepNet in Section 5. All baselines adopt the same branch-wise design as the proposed model: each modality (EEG, EOG, and Leg EMG) is processed by its own dedicated branch, and the resulting branch outputs are concatenated and passed to a shared classification head comprising a GAP layer, an FCL, and a softmax classifier. Only the per-branch feature extractor differs across the three models, which isolates the contribution of the RFEBs and the modality-specific attention of MAF-SleepNet.

The 1D-CNN branch consists of three stacked convolutional blocks [14,15], each containing a causal one-dimensional convolution (kernel size 5, 96 filters), a ReLU activation, layer normalization, and dropout (rate 0.4). The CNN-BiLSTM branch replaces the deeper convolutional stack with a single convolutional block followed by a bidirectional LSTM layer [16] (48 hidden units) and dropout, thereby modeling temporal dependencies recurrently. The Transformer branch first projects each modality to a 48-dimensional representation through a 1×1 convolution and then applies three stacked Transformer encoder blocks [17]; each block comprises a four-head multi-head self-attention sublayer and a position-wise feed-forward network, both wrapped with residual connections and layer normalization.

All baselines were trained under the identical subject-independent protocol with the T1 input (EEG + EOG + Leg EMG), using the Adam optimizer with a learning rate of 0.0001 and left-padding of the variable-length sequences.

References

1. Goldberger, A.L.; Amaral, L.A.; Glass, L.; Hausdorff, J.M.; Ivanov, P.C.; Mark, R.G.; Mietus, J.E.; Moody, G.B.; Peng, C.K.; Stanley, H.E. PhysioBank, PhysioToolkit, and PhysioNet: Components of a New Research Resource for Complex Physiologic Signals. *Circulation* **2000**, *101*, doi:10.1161/01.CIR.101.23.E215.

2. Terzano, M.G.; Parrino, L.; Sherieri, A.; Chervin, R.; Chokroverty, S.; Guilleminault, C.; Hirshkowitz, M.; Mahowald, M.; Moldofsky, H.; Rosa, A.; et al. Atlas, Rules, and Recording Techniques for the Scoring of Cyclic Alternating Pattern (CAP) in Human Sleep. *Sleep Med.* **2001**, *2*, 537–553, doi:10.1016/S1389-9457(01)00149-6.

3. Lee, H.; Li, B.; Huang, Y.; Chi, Y.; Simon, L. NCH Sleep DataBank: A Large Collection of Real-World Pediatric Sleep Studies with Longitudinal Clinical Data (Version 3.1. 0). *PhysioNet* **2021**.

4. Lee, H.; Li, B.; DeForte, S.; Splaingard, M.L.; Huang, Y.; Chi, Y.; Linwood, S.L. A Large Collection of Real-World Pediatric Sleep Studies. *Sci. Data* **2022**, *9*, 1–12, doi:10.1038/s41597-022-01545-6.

5. Wei, X.; Avigdor, T.; Ho, A.; Minato, E.; Garcia-Asensi, A.; Royer, J.; Wang, Y.L.; Travnicek, V.; Schiller, K.; Bernhardt, B.C.; et al. ANPHY-Sleep: An Open Sleep Database from Healthy Adults Using High-Density Scalp Electroencephalogram. *Sci. Data* **2024**, *11*, 1–10, doi:10.1038/s41597-024-03722-1.

6. Hussain, I.; Hossain, M.A.; Jany, R.; Bari, M.A.; Uddin, M.; Kamal, A.R.M.; Ku, Y.; Kim, J.S. Quantitative Evaluation of EEG-Biomarkers for Prediction of Sleep Stages. *Sensors 2022, Vol. 22, Page 3079* **2022**, *22*, 3079, doi:10.3390/S22083079.

7. Aboalayon, K.A.I.; Faezipour, M.; Almuhammadi, W.S.; Moslehpour, S. Sleep Stage Classification Using EEG Signal Analysis: A Comprehensive Survey and New Investigation. *Entropy 2016, Vol. 18, Page 272* **2016**, *18*, 272, doi:10.3390/E18090272.

8. Mendonça, F.; Mostafa, S.S.; Morgado-Dias, F.; Ravelo-García, A.G.; Penzel, T. A Review of Approaches for Sleep Quality Analysis. *IEEE Access* **2019**, *7*, 24527–24546, doi:10.1109/ACCESS.2019.2900345.

9. Pandya, V.; Shukla, U.P.; Joshi, A.M. Novel Features Extraction From EEG Signals for Epilepsy Detection Using Machine Learning Model. *IEEE Sensors Lett.* **2023**, *7*, doi:10.1109/LSENS.2023.3309254.

10. Sharma, M.; Darji, J.; Thakrar, M.; Acharya, U.R. Automated Identification of Sleep Disorders Using Wavelet-Based Features Extracted from Electrooculogram and Electromyogram Signals. *Comput. Biol. Med.* **2022**, *143*, 105224, doi:10.1016/j.compbiomed.2022.105224.

11. Miah, A.S.M.; Hassan, N.; Maniruzzaman, M.; Asai, N.; Shin, J. EMG-Based Hand Gesture Recognition through Diverse Domain Feature Enhancement and Machine Learning-Based Approach. *arXiv Prepr. arXiv2408.13723* **2024**, doi:0.1038/s41598-024-72996-7.

12. Singh, A.K.; Krishnan, S. ECG Signal Feature Extraction Trends in Methods and Applications. *Biomed. Eng. Online* **2023**, *22*, 22, doi:10.1186/s12938-023-01075-1.

13. Wang, L.; Zhou, W.; Chang, Q.; Chen, J.; Zhou, X. Deep Ensemble Detection of Congestive Heart Failure Using Short-Term RR Intervals. *IEEE Access* **2019**, *7*, 69559–69574, doi:10.1109/ACCESS.2019.2912226.

14. Lecun, Y.; Bengio, Y.; Hinton, G. Deep Learning. *Nature* **2015**, *521*, 436–444, doi:10.1038/nature14539.

15. Goodfellow, I.; Bengio, Y.; Courville, A. *Deep Learning*; The MIT Press: Cambridge, MA, USA, 2016; ISBN 978-0262035613.

16. Graves, A.; Schmidhuber, J. Framewise Phoneme Classification with Bidirectional LSTM and Other Neural Network Architectures. *Neural Networks* **2005**, *18*, 602–610, doi:10.1016/J.NEUNET.2005.06.042.

17. Vaswani, A.; Shazeer, N.; Parmar, N.; Uszkoreit, J.; Jones, L.; Gomez, A.N.; Kaiser, L.; Polosukhin, I. Attention Is All You Need. *Adv. Neural Inf. Process. Syst.* **2017**, 5999–6009, doi:10.48550/arXiv.1706.03762.
